# Mitochondria‐Targeting Iron–Carbon Nanozymes Combat Hepatic Ischemia–Reperfusion Injury via the Dual Mechanisms of Respiratory Chain Remodeling and Pyroptosis Inhibition With MRI Monitoring

**DOI:** 10.1002/advs.78277

**Published:** 2026-10-14

**Authors:** Qianying Du, Lian Xu, Tingting Xia, Wenli Zhang, Sijin Chen, Xiyue Rong, Ying Luo, Yi Wang, Jia Liu, Yun Liu, Haitao Ran, Dajing Guo, Jie Xu

**Affiliations:** ^1^ Department of Radiology Second Affiliated Hospital of Chongqing Medical University Chongqing P. R. China; ^2^ Key Laboratory of Intelligent Processing and Applications of Medical Imaging Big Data Chongqing Municipal Health Commission Chongqing P. R. China; ^3^ Engineering Research Center for Fundamental and Translational Intelligent Molecular Imaging Chongqing Municipal Education Commission Chongqing P. R. China; ^4^ Chongqing Key Laboratory of Ultrasound Molecular Imaging & Department of Ultrasound Second Affiliated Hospital of Chongqing Medical University Chongqing P. R. China

**Keywords:** antioxidant therapy, hepatic ischemia–reperfusion injury, nanozymes, pyroptosis inhibition, respiratory chain remodeling

## Abstract

Hepatic ischemia‒reperfusion injury (HIRI) critically compromises liver surgery and transplantation outcomes. Its pathogenesis centers on mitochondrial dysfunction‐driven oxidative stress and sterile inflammation. However, current therapies lack lesion‐specific targeting, fail to suppress ROS generation at the mitochondrial source, and lack integrated theranostic capabilities such as cascade antioxidant activity, anti‐inflammatory effects, and real‐time imaging. To address these limitations, we developed FeCD‐SS31, an integrated nanoplatform composed of a mesoporous Fe_3_O_4_ core conjugated with glutathione/biotin‐derived carbon dots (CDs) and linked to the mitochondrial‐targeting peptide SS31 through an ROS‐responsive bond. The system enabled hepatic mitochondrial targeting and responsive SS31 release, restoring mitochondrial complex integrity to suppress de novo ROS generation. Meanwhile, the Fe_3_O_4_/CDs core exerted cascade enzymatic activities to scavenge existing ROS, achieving dual‐axis oxidative stress inhibition. This synergy blocked the caspase‐1–GSDMD‐N pyroptosis axis and reduced neutrophil infiltration, attenuating inflammation and preserving hepatocytes. These effects alleviated HIRI and improved liver function, while Fe_3_O_4_ superparamagnetism enabled magnetic resonance imaging (MRI) for lesion monitoring. Collectively, this nanoplatform synergistically mitigates HIRI via mitochondrial protection and pyroptosis inhibition, coupled with intrinsic MRI monitoring capability.

## Graphical abstract

A mitochondrial‐targeted FeCD‐SS31 nanoplatform integrates ROS‐responsive SS31 release with cascade antioxidant Fe_3_O_4_/CDs cores to suppress both de novo and existing ROS. By restoring mitochondrial complex integrity and blocking caspase‐1–GSDMD‐N pyroptosis, it reduces neutrophil infiltration and alleviates hepatic ischemia–reperfusion injury, while intrinsic superparamagnetism enables MRI monitoring, offering dual‐axis protection and a promising theranostic strategy.

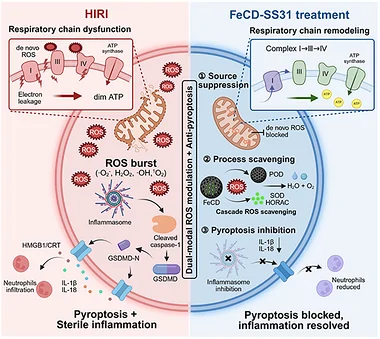

## 1 Introduction

Hepatic resection and transplantation, key surgical interventions for various liver diseases, share a critical clinical challenge: hepatic ischemia‒reperfusion injury (HIRI). This injury follows a “two‐hit” pattern, characterized by initiation during ischemia and exacerbation upon reperfusion, with its core mechanism rooted in the burst generation of reactive oxygen species (ROS) in hepatocytes early after reperfusion [1, 2, 3, 4]. Excessive ROS not only directly induce oxidative stress and cell death but also act as key signaling molecules that activate immune cells and release damage‐associated molecular patterns (DAMPs), thereby triggering and amplifying a robust sterile inflammatory cascade and establishing a vicious cycle of tissue damage [5].

As the primary site of ROS production, mitochondria undergo electron transport chain dysfunction when damaged, resulting in increased electron leakage and subsequent ROS generation [6]. These leaked electrons combine with oxygen molecules to form superoxide anions, which constitute a major source of ROS. The excessive accumulation of ROS not only directly triggers the opening of the mitochondrial permeability transition pore, promoting cytochrome C release and impairing ATP synthesis but also overwhelms the antioxidant defense system of mitochondria, hindering effective clearance. This ongoing process perpetuates oxidative stress, further exacerbating mitochondrial injury and establishing a self‐amplifying vicious cycle. Therefore, impeding this cycle by eliminating ROS and restoring mitochondrial respiratory function represents a crucial therapeutic strategy for HIRI [7, 8, 9].

Currently, the clinical management of HIRI primarily relies on antioxidant agents such as N‐acetylcysteine. However, these drugs are often limited by low bioavailability, poor stability, insufficient targeting capability, and suboptimal therapeutic efficacy [10]. Moreover, the early diagnosis of HIRI still largely depends on conventional biomarkers such as alanine aminotransferase (ALT)/aspartate aminotransferase (AST), which cannot provide visual localization of the lesion and may lead to delayed treatment. Advances in nanotechnology offer new opportunities for theranostic integration [11]: a variety of nano‐antioxidant materials, including ultrasmall copper‐based nanoparticles (NPs) [10], CeO_2_ [12], and Pt‐iNOS@ZIF [13] have demonstrated ROS scavenging capability along with certain imaging functions, showing potential for targeted accumulation and precise treatment at the injury site [14]. As an important branch of these nanomaterials, nanozymes possess advantages such as high stability, low cost, and ease of engineering [15, 16, 17]. A typical example, Fe_3_O_4_ nanozymes, exhibit both catalytic activity and superparamagnetism, enabling their use in magnetic resonance imaging (MRI) [18]. However, under acidic conditions, Fe_3_O_4_ exhibits peroxidase (POD)‐like activity, which may catalyze the conversion of H_2_O_2_ into highly cytotoxic •OH [19]. Without an accompanying cascade antioxidant mechanism, such properties could even exacerbate oxidative damage in HIRI contexts. To overcome the limitations of single nanozyme systems, metal‐free CDs have attracted attention in recent years due to their good biocompatibility and enzyme‐mimicking activities, such as superoxide dismutase (SOD)‐like behavior [20, 21]. In particular, CDs derived from precursors such as glutathione or biotin can scavenge •O_2_^−^ and •OH, offering potential for forming a cascade antioxidant network with Fe_3_O_4_ to improve overall ROS elimination efficiency and reduce secondary free radical risks [22]. However, most existing nano‐antioxidants target accumulated ROS rather than the underlying mitochondrial dysfunction, thereby limiting their ability to achieve sustained suppression of ROS regeneration during HIRI.

The mitochondrial‐targeting peptide SS31 binds to cardiolipin on the inner mitochondrial membrane, stabilizing crista structure and optimizing electron transport chain function [23, 24]. This action reduces mitochondrial ROS production at the source and promotes ATP synthesis, making SS31 an ideal molecular tool for achieving source inhibition [25]. However, as a bioactive peptide, SS31 is susceptible to rapid hydrolysis, fast metabolism, uneven distribution, and quick clearance in vivo, resulting in low bioavailability and significantly limiting its therapeutic efficacy [26, 27]. Furthermore, the pathological mechanism of HIRI extends beyond oxidative stress to inflammatory cell death pathways such as pyroptosis, which triggers neutrophil infiltration and amplifies the inflammatory cascade [28, 29]. Therefore, an ideal intervention strategy for HIRI should systematically address the continuous pathological cascade of “oxidative source suppression–ROS clearance–inflammation regulation,” preferably while also enabling lesion localization and visual monitoring of treatment progress. In summary, a prominent research gap in this field remains the lack of an integrated theranostic platform capable of targeted accumulation at injury sites, simultaneous mitochondrial functional repair (inhibiting ROS generation at the source) and cascade antioxidant activity (clearing existing ROS), coupled with anti‐inflammatory effects and real‐time imaging capability.

Based on this research background, we designed and constructed a novel iron‐carbon composite nanozyme cascade system (FeCD‐SS31) that operates through a multi‐pronged mechanistic framework. Structurally, the system employs mesoporous Fe_3_O_4_ as a carrier loaded with metal‐free CDs synthesized from glutathione and biotin, onto which PEG‐TK‐SS31 is grafted. The ROS‐cleavable TK linker enables targeted release of the SS31 peptide specifically within the diseased region, preventing its premature degradation and ensuring site‐specific action. Mechanistically, FeCD‐SS31 addresses HIRI through three integrated actions: (1) Reduce ROS: ① Source suppression: responsive release of SS31 directly repairs mitochondrial dysfunction and restores respiratory chain integrity, thereby inhibiting de novo ROS generation at its origin. ② Process scavenging: the Fe_3_O_4_/CDs core executes a synergistic enzymatic cascade to efficiently eliminate accumulated ROS. (2) Inflammation and pyroptosis inhibition: transcriptomic and immunofluorescence (IF) analyses confirm that FeCD‐SS31 suppresses the canonical pyroptosis pathway and reduces neutrophil infiltration, blocking both the initiation of damage signals and the subsequent amplification of inflammatory responses. (3) MRI monitoring: leveraging the superparamagnetic properties of Fe_3_O_4_, the platform enables in vivo MRI for lesion dynamic monitoring. In summary, by coordinating mitochondrial repair, catalytic ROS clearance, and immunomodulation within a single theranostic platform with intrinsic imaging capability, FeCD‐SS31 holds promise to provide an integrated solution for HIRI and establish a reference paradigm for managing mitochondrial oxidative stress‐associated ischemia‐reperfusion injury in other organs.

## 2 Results and Discussion

### 2.1 Characterization of Different NPs

FeCD‐SS31 was prepared according to the steps shown in Scheme 1. Mesoporous Fe_3_O_4_ nanocarriers were synthesized by first preparing nonporous NPs via a high‐temperature reaction in ethylene glycol, followed by a pore‐creating hydrothermal treatment. Furthermore, transmission electron microscopy (TEM) characterization revealed that the resulting products exhibited a uniform spherical morphology with clearly visible surface mesopores, indicating their potential as drug carriers (Figure 1A). The CDs, synthesized via a high‐temperature reaction in formamide, exhibited a well‐defined morphology and high size uniformity, as observed by TEM (Figure 1B). The resulting product, designated FeCD‐SS31, was obtained by loading CDs into the mesoporous Fe_3_O_4_ nanostructures (FeCD) and subsequently capping the pores with PEG‐TK‐SS31. The regular morphology and uniform size of the FeCD‐SS31 composite were confirmed by both TEM (Figure 1C) and scanning electron microscopy (SEM) (Figure 1D). High‐angle annular dark field (HAADF) analysis and elemental maps of C, Fe, and O verified the main elemental composition of the FeCD‐SS31 (Figure 1E). Particle size analysis revealed that the diameters of the Fe_3_O_4_, CDs, and final FeCD‐SS31 composite were 144.34 ± 5.42, 5.82 ± 0.14, and 175.26 ± 5.99 nm, respectively (Figure 1F). Similarly, zeta potential measurements via electrophoretic light scattering revealed values of −21.77 ± 2.9 mV for Fe_3_O_4_, −24.84 ± 5.80 mV for CDs, −29.12 ± 1.56 mV for FeCD, and 23.61 ± 1.47 mV for FeCD‐SS31 (Figure 1G).

**SCHEME 1:**
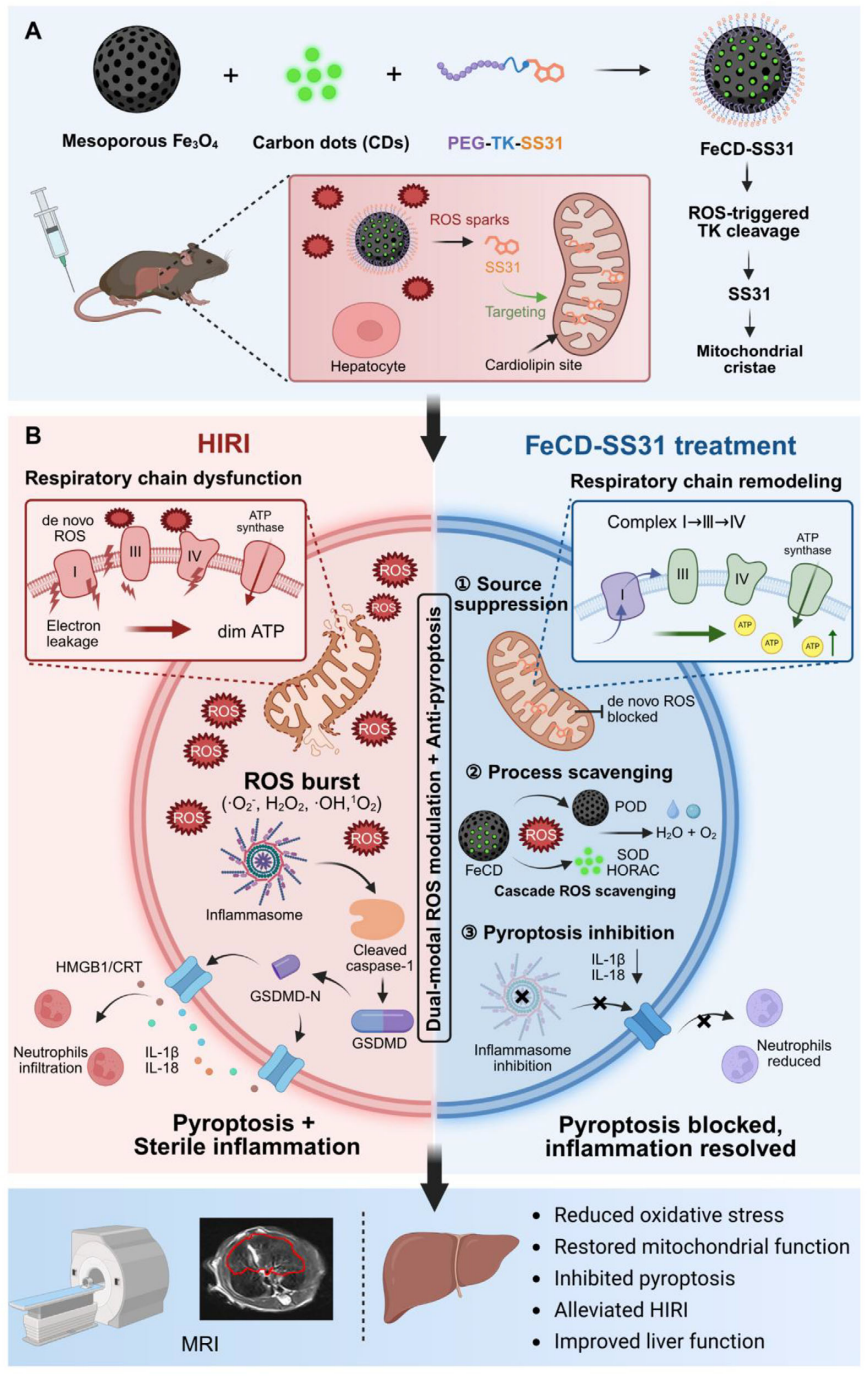
Schematic illustration of the synthesis and mechanism of FeCD‐SS31 in HIRI therapy. (A) Scheme of FeCD‐SS31 synthesis. Upon intravenous administration and hepatic accumulation, the elevated ROS levels in the HIRI microenvironment trigger TK bond cleavage, enabling responsive release and mitochondrial localization of SS31. (B) Mechanism and in vivo MRI monitoring of FeCD‐SS31 for HIRI treatment. The system achieves therapeutic effects through cascade ROS scavenging, mitochondrial respiratory chain repair, and pyroptosis inhibition, leading to reduced oxidative stress, restored mitochondrial function, alleviated HIRI, and improved liver function. Concurrently, the superparamagnetic Fe_3_O_4_ core enables real‐time T_2_‐weighted MRI for noninvasive lesion monitoring.

**FIGURE 1:**
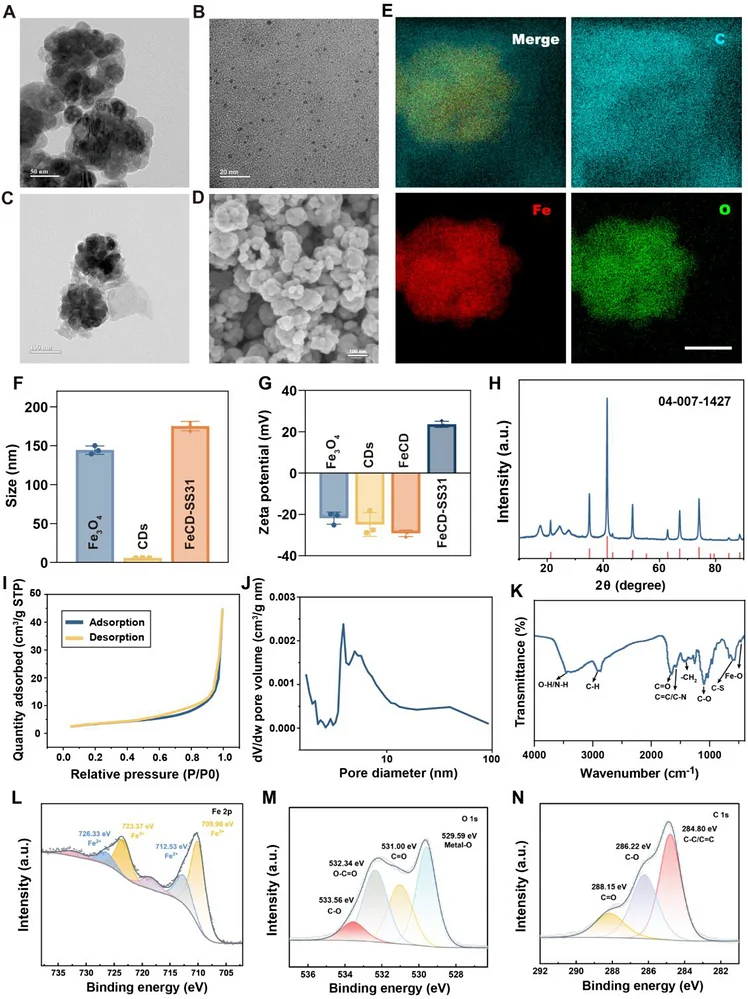
Characterization of the different NPs. TEM images of the different NPs: (A) Fe_3_O_4_, (B) CDs, and (C) FeCD‐SS31. (D) SEM image of FeCD‐SS31. (E) HAADF and elemental mapping of FeCD‐SS31 (scale bar: 50 nm). (F) Diameters and (G) zeta potentials of the different NPs (*n* = 3). (H) XRD patterns of Fe_3_O_4_. (I) N2 adsorption‒desorption isotherm and (J) pore size distribution of Fe_3_O_4_. (K) FTIR spectra of FeCD‐SS31. XPS spectra of Fe 2p (L), O1s (M), and C1s (N) with peak fitting.

The X‐ray diffraction (XRD) pattern confirmed the successful synthesis of phase‐pure Fe_3_O_4_ NPs. All diffraction peaks were perfectly indexed to the cubic spinel structure of magnetite (JCPDS no. 04‐007‐1427). The sharp and intense peaks suggested that the obtained product was well crystallized (Figure 1H). The magnetic properties of the FeCD‐SS31 NPs were characterized using a vibrating sample magnetometry (VSM). Hysteresis loop measurements of FeCD‐SS31 revealed no detectable remanent magnetization (Figure S1), demonstrating its superparamagnetic behavior at room temperature. N_2_ adsorption‒desorption analysis revealed that the as‐synthesized Fe_3_O_4_ exhibited a type IV isotherm with an H3 hysteresis loop (P/P_0_ = 0.4–1.0), characteristic of a mesoporous structure (Figure 1I). The material possessed an average pore size of 8.74 nm and a pore volume of 0.024 cm^3^·g^−^^1^, which are suitable for accommodating CDs (Figure 1J). The Fourier transform infrared (FT‐IR) spectrum of FeCD‐SS31 is presented in Figure 1K. A broad overlapping band in the 3455–3310 cm^−^^1^ region was attributed to O–H and N–H stretching vibrations, originating from surface hydroxyl groups on the CDs and amide groups from the polypeptide. The absorption peaks at 2907 and 2868 cm^−^^1^ were assigned to C‐H stretching vibrations of aliphatic chains present in the CDs and PEG‐TK‐SS31. The peak at 1651 cm^−^^1^ was primarily associated with C = O stretching vibrations from surface carbonyls on the CDs, with a potential contribution from the amide C = O stretching of the polypeptide. The absorption at 1578 cm^−^^1^ corresponded to aromatic C = C stretching and amide C‐N stretching vibrations. Peaks at 1435 and 1253 cm^−^^1^ were characteristic of methylene bending vibrations and C‐N stretching vibrations, respectively. The strong absorption near 1093 cm^−^^1^ stemmed from C‐O stretching vibrations of various oxygen‐containing groups in the composite. The absorption band observed at 605.68 cm^−^^1^ can be assigned to the C–S stretching vibration of PEG‐TK‐SS31. Critically, the weak but distinct peak at 461 cm^−^^1^, characteristic of the Fe–O lattice vibration of Fe_3_O_4_, suggested effective surface coating by CDs and PEG‐TK‐SS31, resulting in a high loading that largely masked the underlying magnetic core. X‐ray photoelectron spectroscopy (XPS) was used to analyze the valence states of Fe, C, O and N (Figure S2). The iron 2p3/2 spectrum was deconvoluted into two characteristic bands at 709.96 and 712.53 eV, which were indexed to Fe^2+^ and Fe^3+^, respectively (Figure 1L). As shown in Figure 1M, the peak at 529.59 eV in the O 1s spectrum corresponded to metal‐O, the peak at 531.00 eV corresponded to C═O, the peak at 532.34 eV corresponded to O─C═O, and the peak at 533.56 eV corresponded to C─O. The peak at 284.8 eV in the C 1s spectrum corresponded to C─C/C═C, the peak at 286.22 eV corresponded to C─O, and the peak at 288.15 eV corresponded to C═O (Figure 1N). The peaks at 399.65 and 401.76 eV in the N 1s spectrum represented C─N and N─H, respectively (Figure S3). XPS analysis provided evidence for the successful synthesis of the FeCD‐SS31 nanozyme. Specifically, the metal–O peak in the O 1s spectrum at 529.59 eV directly confirmed the formation of Fe–O bonds, whereas the characteristic peaks in the C 1s and N 1s spectra corresponded to the functional groups of the CD framework and the SS31 peptide, respectively. Together with the results from FT‐IR and other techniques, these data collectively demonstrate the successful synthesis of FeCD‐SS31. The iron content of FeCD‐SS31 was analyzed using Inductively coupled plasma‒optical emission spectrometry (ICP‒OES), which yielded a value of 0.154 mg mg^−^^1^. The encapsulation efficiency (EE) and loading capacity (LC) of the CDs were 81.02% and 19.06%, respectively, as determined by UV–vis spectrophotometry.

A dose‐response release assay was performed to evaluate ROS‐triggered SS31 release. FeCD‐SS31 (FITC‐labeled SS31) were encapsulated in dialysis bags and exposed to 0–200 µm H_2_O_2_. Released SS31 was quantified by fluorescence intensity. A marked increase in release was observed above 60 µm H_2_O_2_, with a plateau at approximately 120 µm, indicating near‐complete release (Figure S4). The threshold was found to be within the pathophysiological ROS range reported for HIRI, confirming that physiological ROS levels are sufficient for effective system activation.

The aforementioned analyses collectively corroborated the successful construction of the FeCD‐SS31 nanozymes, providing unequivocal evidence for their intended structure.

### 2.2 In Vitro MRI

Due to its excellent soft tissue resolution and deep tissue penetration capability, MRI has become a widely adopted imaging examination method in the clinical diagnosis of liver diseases. As illustrated in Figure S5, the T_2_‐weighted MR signal intensity decreased in a concentration‐dependent manner with increasing NPs concentration. Furthermore, a strong linear correlation was observed between the reciprocal of the T_2_ relaxation time (1/T_2_) and the corresponding iron concentration, yielding a r_2_ of 120.91 mm ^−^^1^·s^−^^1^, as demonstrated in Figure S6. These results demonstrate that the NPs serve as an efficient T_2_ MRI contrast agent, providing satisfactory imaging performance even at low concentrations.

### 2.3 The Cytotoxicity, Intracellular Endocytosis of FeCD‐SS31 and the Establishment of an In Vitro HIRI Model

#### 2.3.1 In Vitro Cytotoxicity Evaluation

The toxicities of the different NPs to AML‐12 cells were determined by CCK‐8 assays. AML‐12 cells were incubated with various NPs (Fe_3_O_4_, FeCD, and FeCD‐SS31) at concentrations ranging from 50 to 300 µg/mL. The results indicated that cell viability remained above 70% even at the highest concentration; the IC50 values for Fe_3_O_4_, FeCD, and FeCD‐SS31 were determined to be 1510, 1045, and 688.3 µg/mL, respectively, demonstrating the favorable biocompatibility of the synthesized nanozymes in vitro (Figure 2B). Based on these findings, a concentration of 200 µg/mL, which supported a cell viability exceeding 80%, was selected for all subsequent experiments.

**FIGURE 2:**
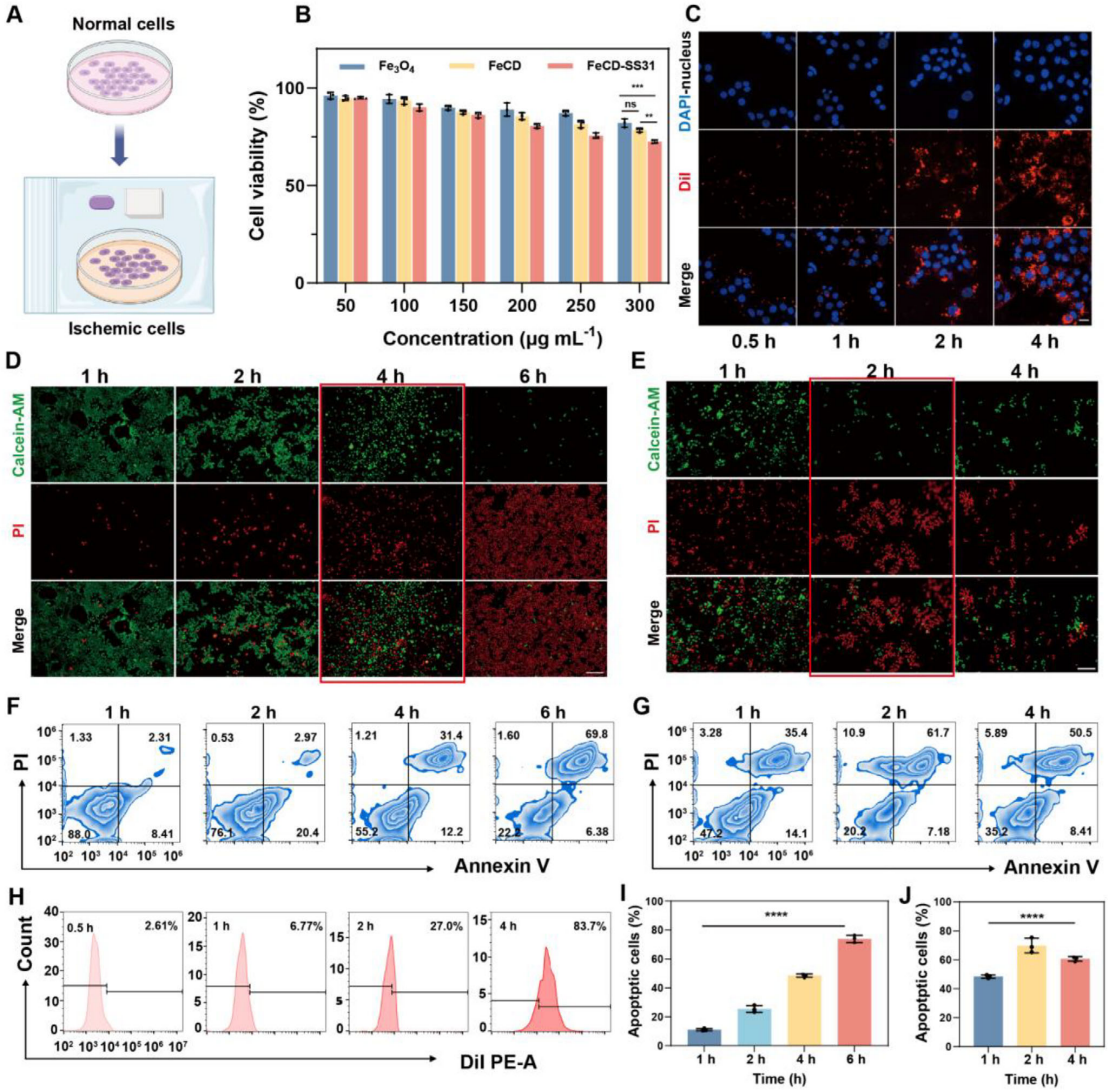
Intracellular endocytosis, the cytotoxicity of FeCD‐SS31, and the establishment of an in vitro HIRI model. (A) Schematic illustration of the in vitro ischemic model established via the hypoxia chamber method. (B) Viability of AML‐12 cells after incubation with different concentrations of Fe_3_O_4_, FeCD, and FeCD‐SS31 for 12 h (*n* = 3). (C) CLSM images of the intracellular endocytosis of FeCD‐SS31@DiI (scale bar: 20 µm). (D,E) CLSM images of live and dead AML‐12 cells after different ischemia/reperfusion times, in which reperfusion was performed after 4 h of ischemia (scale bars: 100 µm). and (F,G) the corresponding FCM analysis of live cells and apoptotic cells. (H) FCM analysis of the intracellular endocytosis of FeCD‐SS31@DiI (*n* = 3). (I,J) Quantitative analysis of live cells and apoptotic cells. In panels (B), (I,J), the data are presented as the mean ± SD, and the *p*‐values were calculated by ANOVA. ∗ *p* < 0.05, ∗∗ *p* < 0.01, ∗∗∗ *p* < 0.001, ∗∗∗∗ *p* < 0.0001; ns, not significant.

#### 2.3.2 Intracellular Endocytosis

Given that FeCD‐SS31 was developed in this study to alleviate HIRI, it was essential to first verify its cellular uptake by hepatocytes (AML‐12). Confocal laser scanning microscopy (CLSM) revealed a time‐dependent increase in the red fluorescence (FL) signals of FeCD‐SS31@DiI around DAPI‐stained nuclei. The FL intensity peaked at 4 h (Figure 2C). The observed FL kinetics provide clear evidence for the dynamic uptake of FeCD‐SS31@DiI in hepatocytes. Flow cytometry (FCM) was used to quantify the intracellular uptake efficiency of FeCD‐SS31@DiI in AML‐12 cells, which was approximately 83.7% after a 4‐h incubation, which is consistent with the CLSM observations (Figure 2H). Collectively, these findings demonstrate the high capacity of AML‐12 cells for FeCD‐SS31@DiI internalization.

#### 2.3.3 Establishment of an In Vitro HIRI Model

To established in vitro ischemic model, we used a hypoxia chamber (containing a gas‐generating pack and an indicator) to set up the cell model, ensuring a stable and controlled low‐oxygen environment (Figure 2A). Based on the results of live/dead staining and apoptotic FCM, the cell death rates under ischemia were 11.1% ± 0.79% (1 h), 25.46% ± 2% (2 h), 43.6% ± 0.8% (4 h), and 73.89% ± 2.48% (6 h) (Figure 2D,F,I). Given the excessively high mortality after 6 h of hypoxia, a 4‐h ischemia period was selected for the subsequent reperfusion study to enable observation of the reperfusion process. Further assessment during reperfusion revealed that peak cell death occurred at the 2 h time point (Figure 2E,G,J). Accordingly, a 2‐h reperfusion duration was chosen to establish the in vitro HIRI model.

### 2.4 Analysis of In Vitro ROS Scavenging and Mitochondrial Targeting

The FeCD‐SS31 nanozyme possesses a unique multienzyme profile (POD, SOD, HORAC, and singlet oxygen (^1^O_2_) scavenging; Figure 3A), which allows it to execute a protective antioxidant cascade. Rather than producing secondary radicals such as •OH, a typical limitation of single POD‐mimetic enzymes, our nanozyme cooperatively neutralizes a wide range of ROS and ultimately converts them into water and oxygen. This capacity for synergistic and cascade reactions makes it particularly effective against the complex oxidative stress found in pathological conditions [30].

**FIGURE 3:**
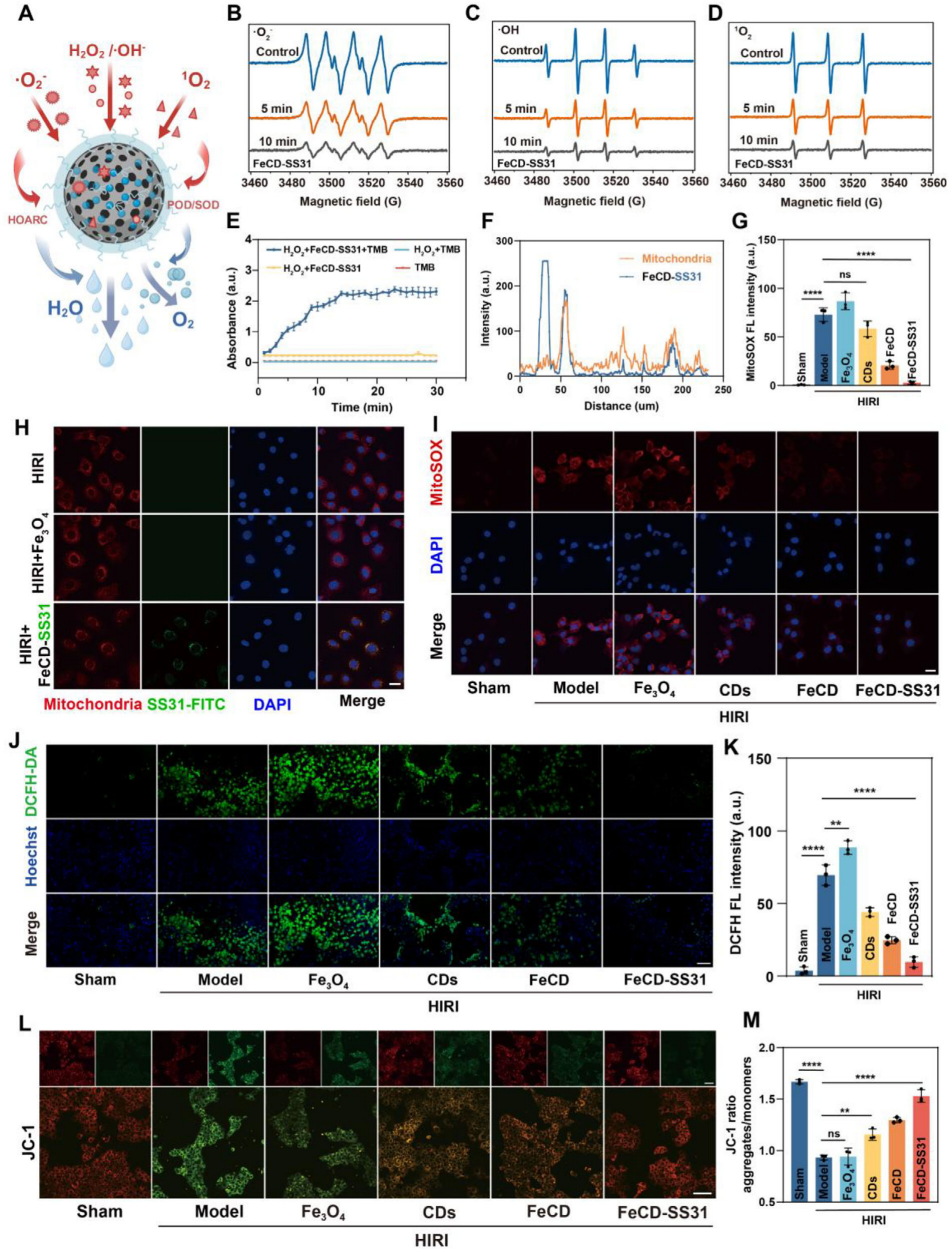
ROS Scavenging In Vitro and Mitochondrial Targeting. (A) Multienzyme‐mimicking activities of FeCD‐SS31 nanozymes. ESR spectra of the time‐dependent scavenging of (B) •O_2_^−^, (C) •OH, and (D) ^1^O_2_ by FeCD‐SS31. (E) POD‐like activity of FeCD‐SS31. (F) Analysis of the colocalization of FeCD‐SS31 with mitochondria. (G) Corresponding quantitative analysis of MitoSOX expression (*n* = 3). (H) Representative CLSM images showing the colocalization of FeCD‐SS31 with mitochondria (scale bars: 25 µm). (I) Representative CLSM images of MitoSOX Red staining (scale bars: 25 µm). (J) DCFH‐DA staining images of AML‐12 cells with different treatments (scale bar: 100 µm) and (K) corresponding quantitative analysis (*n* = 3). (L) Representative CLSM images of JC‐1 (scale bars: 100 µm) and (M) corresponding quantitative analysis (*n* = 3). In panels (E), (G), (K) and (M), the data are presented as the mean ± SD, and the *p*‐values were calculated by ANOVA. ∗ *p* < 0.05, ∗∗ *p* < 0.01, ∗∗∗ *p* < 0.001, ∗∗∗∗ *p* < 0.0001; ns, not significant.

A classic 3,3',5,5'‐Tetramethylbenzidine (TMB) colorimetric assay confirmed the POD‐like activity of FeCD‐SS31 nanozymes, which catalyzed the H_2_O_2_‐dependent oxidation of TMB, yielding a time‐dependent increase in the absorbance characteristic of blue oxTMB. This catalytic response, which was absent in the control groups (Figure 3E and Figure S7), demonstrated the effective POD‐mimetic function of the nanozymes. To further quantify the catalytic efficiency of the POD‐like activity, we performed steady‐state kinetic assays by varying the TMB concentration at a fixed H_2_O_2_ concentration. The kinetic data were fitted to the Michaelis–Menten model, yielding the following parameters: K_m_ = 4.026 mm, V_max_ = 14.38 mm·min^−^^1^, and turnover frequency (TOF) = 0.523 min^−^^1^. These results provide quantitative evidence for the substrate affinity and catalytic turnover capacity of the FeCD‐SS31 nanozymes, further supporting their robust POD‐mimetic activity (Figure S8).

Superoxide anions (•O_2_^−^), as primary ROS generated from sources such as the electron transport chain, can directly inflict cellular damage or be converted into more reactive species, such as the highly destructive •OH. The •OH radical instantly attacks biological macromolecules, causing severe structural and functional impairments. Electron paramagnetic resonance (ESR) spectroscopy, in which 5,5‐dimethyl‐1‐pyrroline N‐oxide (DMPO) was used as a spin trap, demonstrated the efficacy of FeCD‐SS31 nanozymes in scavenging both •O_2_^−^ and •OH. As shown in Figure 3B,C, a significant time‐dependent (5 and 10 min) decrease in ESR signal intensity was observed upon treatment with the nanozymes, confirming their dual SOD‐like activity and HOARC capacity. To further quantitatively evaluate the SOD‐mimetic activity, we determined the apparent Michaelis constant (K_m_) of FeCD‐SS31 using xanthine as the substrate in a xanthine system. The apparent K_m_ was calculated based on the inhibition rate of superoxide‐mediated chromogenic signal and was found to be 1.128 mm (Figure S9). This apparent value provides a useful indicator of the substrate affinity of FeCD‐SS31 toward superoxide anions, further supporting its efficient SOD‐like activity.

The ^1^O_2_‐scavenging ability of FeCD‐SS31 was also directly confirmed by ESR spectroscopy using TEMP as a specific spin trap. The observed significant reduction in the characteristic TEMP‐^1^O_2_ signal intensity upon FeCD‐SS31nanozymes treatment demonstrated its powerful deactivation capability. This was attributed to the catalytic function of the nanozymes, providing a favorable surface for the physical quenching of ^1^O_2_, promoting its return to the ground state and thereby mitigating its oxidative damage (Figure 3D).

SS31 is a mitochondria‐targeting peptide that specifically interacts with cardiolipin, an essential phospholipid exclusively located in the inner mitochondrial membrane, and plays a critical role in crista formation [31]. This binding enables SS31 to inhibit the oxidation of both cardiolipin and cytochrome c, thereby preserving crista integrity. Additionally, SS31 stabilizes the electron transport chain, facilitating efficient electron transfer and oxidative phosphorylation while reducing mitochondrial ROS generation. Through its electrostatic and hydrophobic interactions with cardiolipin, SS31 further enhances ATP production [32]. To verify the mitochondrial targeting ability of our engineered nanozyme, we fluorescently labeled them with SS31 and performed a confocal microscopy‐based colocalization analysis (Figure 3F,H). Pearson correlation analysis was performed to evaluate the colocalization between SS31 and mitochondria. The analysis yielded a Pearson correlation coefficient of r = 0.70021393, indicating relatively strong colocalization of SS31 with mitochondria. The pronounced colocalization of SS31 FL with mitochondrial signals confirmed the effective accumulation of the nanozyme in the mitochondria.

The levels of mitochondrial superoxide and intracellular ROS collectively reflect the overall oxidative stress status in the cellular environment [33]. To evaluate this, mitochondrial superoxide and intracellular ROS levels in AML‐12 cells were detected using MitoSOX Red Mitochondrial Superoxide Indicator (Figure 3G,I) and DCFH‐DA (green FL), respectively. Compared with the control group, the HIRI group exhibited significantly stronger red FL intensity in the mitochondria, indicating that increased mitochondrial superoxide accumulation was induced by HIRI. Notably, compared with the HIRI group, the Fe_3_O_4_ group demonstrated even more intense red FL. Rather than providing protection, Fe_3_O_4_ alone exacerbated oxidative damage, which is consistent with its documented function as a pure POD mimetic that converts H_2_O_2_ into harmful •OH radicals, thereby failing to alleviate and even amplifying cellular injury. In contrast, CDs suppressed mitochondrial superoxide release via their SOD‐like activity, whereas SS‐31 directly reduced de novo ROS generation at the source. Moreover, the HORAC activity of the CDs effectively complemented the POD‐like function of Fe_3_O_4_, establishing a catalytic cascade that synergistically eliminated •OH. A key design feature involved engineering the FeCD with surface‐anchored SS31 peptides via a ROS‐cleavable TK linker. This configuration enabled FeCD‐SS31 to concurrently consume ROS and, in response, trigger the localized release of encapsulated CDs and SS31 peptides at the target site, thereby further enhancing its integrated antioxidant capacity. A similar trend was observed in the green FL measurements, corroborating the coordinated action of the nanozyme components (Figure 3J,K). Furthermore, the mitochondrial membrane potential was assessed using a JC‐1 FL assay. In this assay, JC‐1 aggregates (red FL) indicate normal mitochondrial polarization, whereas JC‐1 monomers (green FL) indicate mitochondrial depolarization. As shown in Figure 3L,M, compared with the cells in the other treatment groups, the AML‐12 cells treated with FeCD‐SS31 exhibited a mitochondrial membrane potential closest to that of the cells in the sham group. These results suggest that FeCD‐SS31 nanozymes have the most pronounced protective effect on mitochondrial membrane integrity.

In summary, the engineered FeCD‐SS31 nanozyme integrates multiple antioxidant activities into a single cascade‐driven system. Unlike conventional single‐enzyme mimics, it cooperatively neutralizes a broad spectrum of ROS without generating secondary radicals, ultimately converting them into water and oxygen. Mitochondrial targeting, which is conferred by the SS31 peptide, was confirmed via FL colocalization, ensuring precise accumulation at the site of oxidative injury. The results of functional assays demonstrated that FeCD‐SS31 effectively quenched mitochondrial superoxide, intracellular ROS, and hydroxyl radicals while preserving the mitochondrial membrane potential. Notably, its multi‐enzyme cascade and responsive release mechanism worked synergistically to mitigate oxidative damage, outperforming individual components such as Fe_3_O_4_ or CDs alone. These findings establish FeCD‐SS31 as a potent and mitochondrially targeted nanozyme capable of counteracting complex oxidative stress under pathological conditions.

### 2.5 RNA Sequencing to Explore the Biological Mechanisms of FeCD‐SS31

To elucidate the biological pathways underlying the anti‐inflammatory effects of FeCD‐SS31, transcriptomic profiling of murine liver tissues was conducted across I/R‐induced and FeCD‐SS31‐treated conditions. Comparative analysis revealed significant intergroup disparities in gene expression patterns, as visualized in the differential gene heatmap (Figure 4A). Moreover, the absence of significant intragroup variations confirmed the reliability of the data. Principal component analysis (PCA) further revealed clear transcriptional distinctions between groups alongside high reproducibility among biological replicates (Figure 4B). Through volcano plot analysis employing thresholds of |log_2_ (fold change) | > 1 and −log_10_ (*p*‐value) > 1.3, we identified 1,080 significantly upregulated and 955 downregulated genes in FeCD‐SS31‐treated samples relative to those in HIRI controls (Figure 4C). Gene Ontology (GO) enrichment analysis of these differentially expressed genes revealed three functional categories: biological processes, cellular components, and molecular functions (Figure 4D). Among the significantly enriched terms, oxidoreductase activity (GO: 0016491) was particularly noteworthy. Subsequent heatmap visualization revealed upregulated expression levels of multiple oxidoreductases in the mitochondrial respiratory chain following FeCD‐SS31 treatment (Figure 4E). The suppression of redox stress in the mitochondrial respiratory chain via the robust antioxidant capacity of FeCD‐SS31 may explain these findings. Concurrent Kyoto Encyclopedia of Genes and Genomes (KEGG) analysis revealed its involvement in the regulation of several inflammatory pathways, particularly the NOD‐like receptor signaling cascade (Figure 4F). A more detailed analysis of the NOD‐like receptor signaling pathway was conducted, with a |Log_2_FC| > 2 set as the threshold for statistical significance. We observed significant downregulation of *nod2*, a gene critical for inflammasome assembly. This downregulation was predicted to attenuate caspase‐1 activation, leading to reduced levels of cleaved caspase‐1 and GSDMD‐N, thereby suppressing canonical pyroptosis (Figure 4G). GSEA analysis showed that, compared with the treatment group, the model group exhibited significantly positive enrichment of pyroptosis‐related gene sets, confirming that the canonical pyroptosis pathway was suppressed by nanozyme treatment (Figure S10). Consequently, the release of proinflammatory cytokines such as IL‐1β and IL‐18 was diminished. The mechanistic impact of FeCD‐SS31 on this pathway will be further examined using integrated cellular and animal studies. Moreover, we have performed GO enrichment analysis and generated a bubble plot. The results show that terms associated with energy metabolism are significantly enriched in the treatment group compared with the model group, indicating that nanozyme treatment enhances mitochondrial energy metabolism (Figure S11).

**FIGURE 4:**
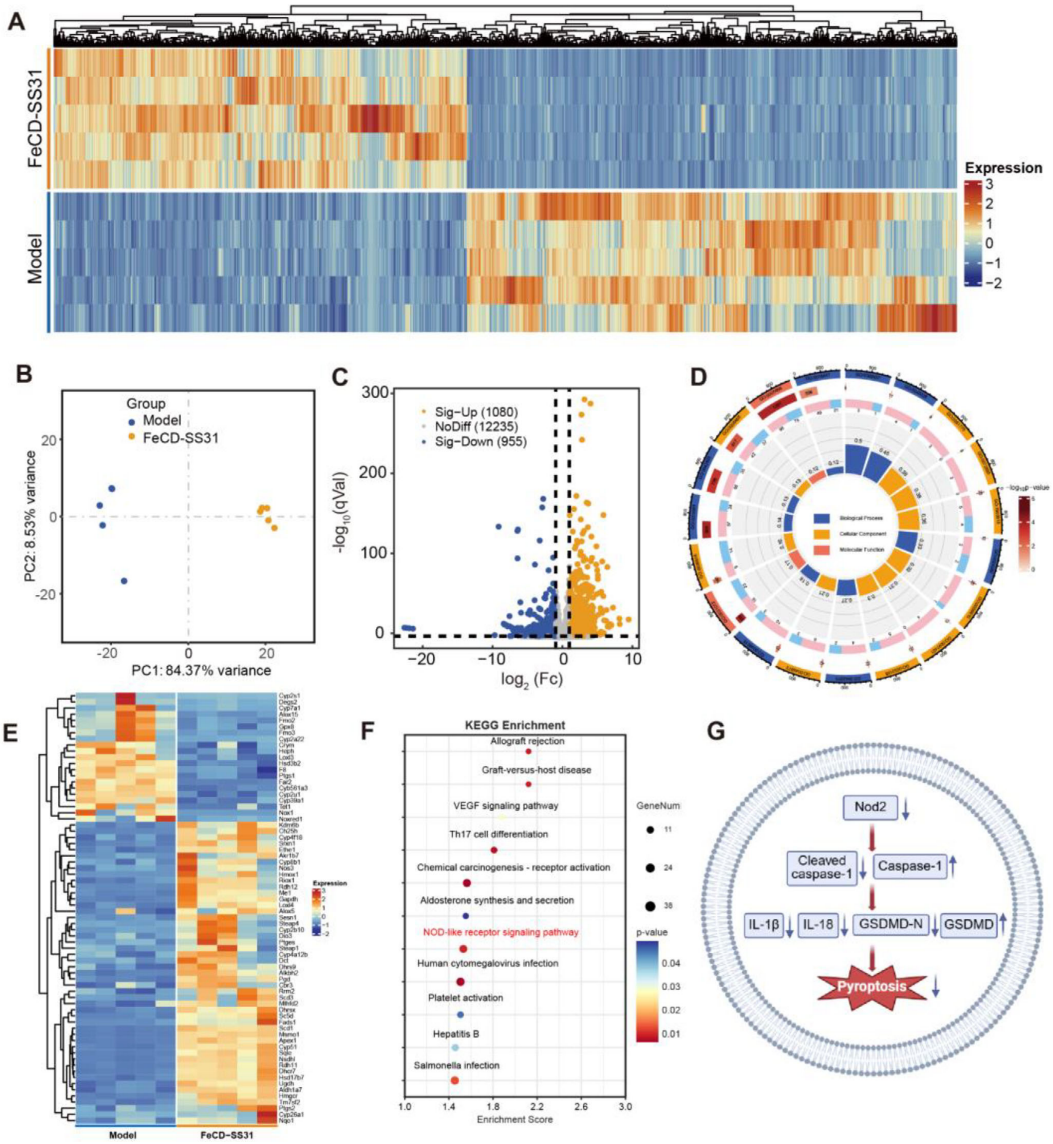
RNA sequencing to explore the biological mechanisms of FeCD‐SS31. (A) Heatmap of gene expression under different treatments. (B) PCA of the HIRI+FeCD‐SS31 and HIRI groups. (C) Volcano plots of all genes in the HIRI+FeCD‐SS31 and HIRI groups. The yellow dots represent the upregulated differentially expressed genes, the blue dots represent the downregulated differentially expressed genes, and the gray dots represent the nondifferentially expressed genes. (D) GO enrichment of differentially expressed genes in the HIRI+FeCD‐SS31 and HIRI groups. (E) Heatmap of genes with GO: 0016491 in the GO analysis. (F) KEGG enrichment of differentially expressed genes in the HIRI+FeCD‐SS31 and HIRI groups. (G) Schematic diagram of how FeCD‐SS31 affects the classical cellular pyroptosis pathway.

### 2.6 Mechanisms of FeCD‐SS31 in the Treatment of HIRI In Vitro and In Vivo

#### 2.6.1 In Vitro and In Vivo Investigation of Pyroptosis Inhibition by FeCD‐SS31

Based on our previous transcriptomic analysis, we have provided preliminary evidence that the constructed nanozyme alleviates HIRI by suppressing the caspase‐1‐mediated canonical pyroptosis pathway. Activation of the canonical pyroptosis pathway is characterized primarily by caspase‐1 activation, increased levels of cleaved caspase‐1, cleavage of GSDMD, cell swelling and rupture, and the subsequent release of numerous inflammatory factors. To further validate the inhibitory effect of the nanozyme on pyroptosis at the cellular level, we first performed IF staining to measure the expression of calreticulin (CRT), high‐mobility group box1 (HMGB1), and cleaved caspase‐1 [34, 35].

Under physiological conditions, both CRT and HMGB1 exhibit well‐defined intracellular localization. During pyroptosis, their normal distribution patterns are disrupted: CRT becomes detectable in the cytoplasm, whereas HMGB1 is translocated from the nucleus and ultimately released into the extracellular space. Thus, the aberrant localization and release of CRT and HMGB1 can serve as indirect indicators of pyroptosis [36].

As shown in Figure 5A and Figure S12, HMGB1 was predominantly expressed in the nucleus in the sham group. Following HIRI, the FL intensity of HMGB1 in the nucleus decreased significantly, suggesting its nuclear translocation and association with pyroptosis. Treatment with Fe_3_O_4_ and CDs did not lead to significant recovery of nuclear HMGB1 FL intensity, indicating limited ameliorative effects on pyroptosis. In contrast, treatment with both FeCD and FeCD‐SS31, particularly the latter, markedly restored the nuclear HMGB1 FL intensity, demonstrating that these nanozymes effectively inhibited HMGB1 release into the extracellular space, thereby alleviating pyroptosis. The IF results for CRT are shown in Figure 5B and Figure S13. In the sham group, CRT expression in the cytoplasm was minimal; after IR treatment, its cytoplasmic expression significantly increased. Consistent with the HMGB1 findings, treatment with Fe_3_O_4_ or CDs alone did not notably ameliorate CRT accumulation in the cytoplasm. In contrast, both the FeCD and FeCD‐SS31 treatments significantly reduced the abnormal cytoplasmic accumulation of CRT, with FeCD‐SS31 having the most pronounced effect. These results further confirm that FeCD‐SS31 effectively inhibits signaling events associated with pyroptosis. Cleaved caspase‐1 is a key executor of pyroptosis and a specific marker of the canonical pathway. IF analysis demonstrated that ischemia‒reperfusion injury significantly increased cleaved caspase‐1 FL intensity, whereas FeCD‐SS31 treatment effectively reduced this signal to levels approaching those in the sham group. This pattern confirms that FeCD‐SS31 substantially inhibits the canonical pyroptosis pathway (Figure 5C and Figure S14).

**FIGURE 5:**
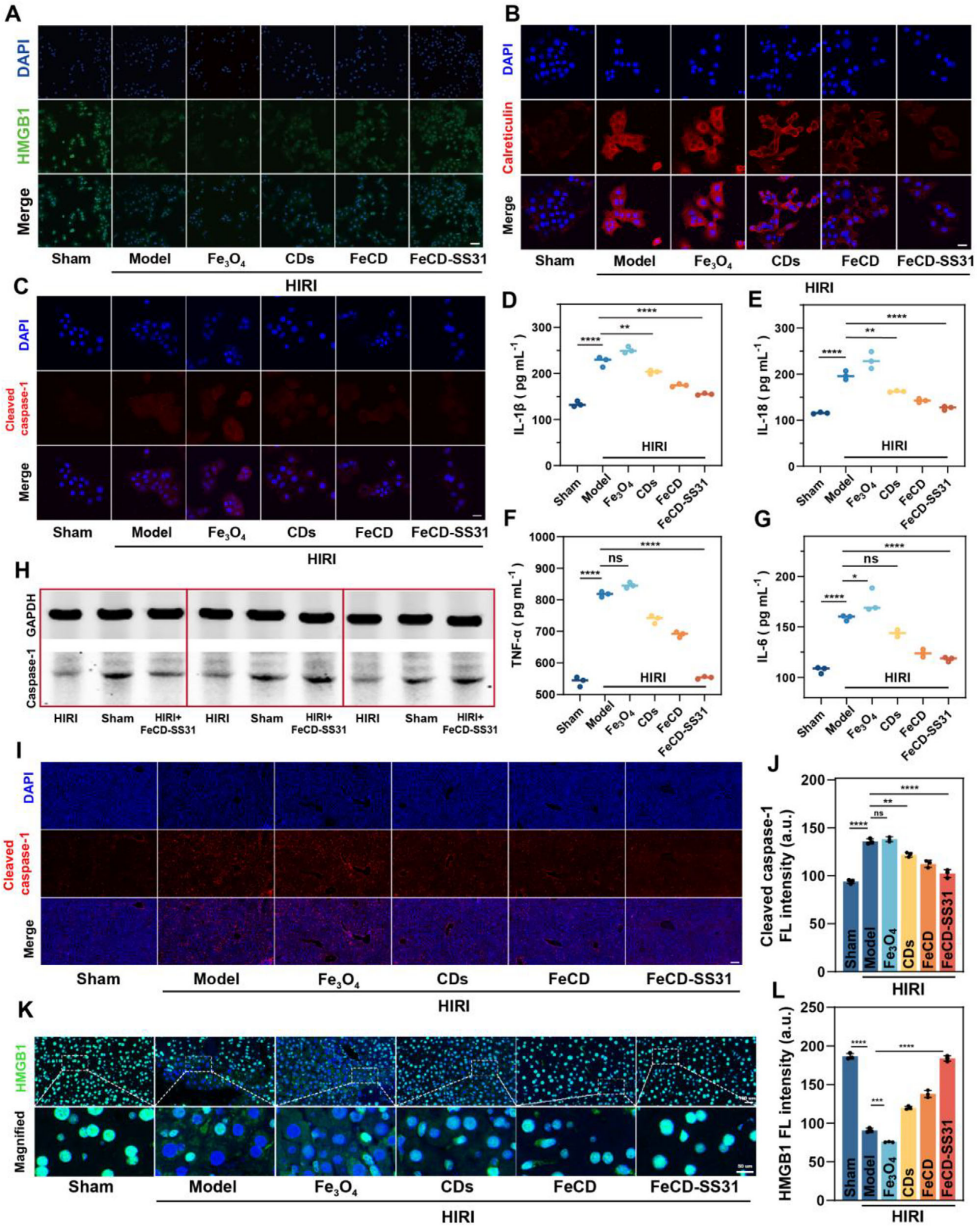
Mechanisms of FeCD‐SS31 in the treatment of HIRI in vitro and in vivo. CLSM images of (A) HMGB1 expression, (B) CRT expression, (C) cleaved caspase‐1 expression in AML‐12 cells (scale bars: 25 µm). The levels of (D) IL‐1β, (E) IL‐18, (F) TNF‐α, and (G) IL‐6 were measured in the cell supernatant after various treatments (*n* = 3). (H) WB analysis of caspase‐1 in vivo. IF images of (I) cleaved caspase‐1(scale bar: 100 µm) and (K) HMGB1 expression in liver tissue after different treatments. Semiquantitative FL intensity analysis of (J) cleaved caspase‐1 and (L) HMGB1 staining (*n* = 3). In panels (D,E,G,H,J,L), the data are presented as the mean ± SD, and the *p*‐values were calculated by ANOVA. ∗ *p* < 0.05, ∗∗ *p* < 0.01, ∗∗∗ *p* < 0.001, ∗∗∗∗ *p* < 0.0001; ns, not significant.

During pyroptosis, released CRT and HMGB1 act as DAMPs that activate numerous immune cells and stimulate the secretion of various inflammatory cytokines, thereby exacerbating local inflammation [37]. To verify this mechanism, we measured the concentrations of IL‐18, IL‐1β, TNF‐α, IFN‐γ, IL‐12, IL‐1, and IL‐6 in cell culture supernatants using enzyme‐linked immunosorbent assays (ELISAs). The results revealed significantly elevated levels of these cytokines in the HIRI group, with further increases in the levels of some inflammatory factors in the Fe_3_O_4_ treatment group, which may have been due to increased •OH generation following Fe_3_O_4_ administration. In contrast, a sequential reduction in cytokine concentrations was observed in the CDs, FeCD, and FeCD‐SS31 treatment groups, with FeCD‐SS31 exhibiting the most pronounced anti‐inflammatory effect, indicating effective suppression of pyroptosis‐related inflammation (Figure 5D–G and Figure S15).

To further validate the inhibitory effect of the nanozyme on pyroptosis in vivo, we performed Western blot (WB) analysis on liver tissues from the HIRI, sham, and FeCD‐SS31‐treated groups to assess caspase‐1 and GSDMD expression. During pyroptosis, caspase‐1 is activated and cleaved into its active form, resulting in a decrease in full‐length caspase‐1 protein levels. Concurrently, activated caspase‐1 cleaves GSDMD to generate the N‐terminal pore‐forming fragment (GSDMD‐N), leading to a decrease in full‐length GSDMD and an increase in GSDMD‐N. As shown in Figure 5H, Figures S16 and S17, compared with the sham group, the HIRI group exhibited significantly reduced full‐length caspase‐1 and full‐length GSDMD, accompanied by a marked increase in GSDMD‐N, confirming the occurrence of canonical pyroptosis. In contrast, FeCD‐SS31 treatment restored caspase‐1 and full‐length GSDMD levels to those comparable to the sham group, while GSDMD‐N expression was maintained at sham levels. These results demonstrate that FeCD‐SS31 effectively suppresses aberrant caspase‐1 activation and subsequent GSDMD cleavage, thereby inhibiting pyroptosis in vivo.

Consistent with these findings, we performed IF staining for HMGB1, CRT, and cleaved caspase‐1 in liver tissues from the different treatment groups. As shown in Figure 5I–L and Figure S18, the expression patterns of these markers at the tissue level aligned well with the cellular IF observations, collectively demonstrating that FeCD‐SS31 effectively inhibits pyroptosis in vivo.

Overall, guided by preliminary transcriptomic analysis, we conducted corresponding in vitro and in vivo experiments to validate the underlying mechanism. The alterations in the subcellular localization of HMGB1 and CRT indirectly confirmed that FeCD‐SS31 effectively inhibited pyroptosis. Moreover, changes in the expression of caspase‐1 and its cleaved form directly reflected the inhibitory effect of the nanozymes on the canonical pyroptosis pathway. ELISA measurements of inflammatory cytokines further demonstrated that FeCD‐SS31 significantly alleviated pyroptosis‐associated inflammation. Collectively, these results indicate that the FeCD‐SS31 nanozymes suppress the canonical pyroptosis pathway at both the cellular and animal levels, thereby mitigating inflammatory damage related to HIRI.

#### 2.6.2 In Vivo Restoration of the Mitochondrial Respiratory Chain by FeCD‐SS31 Nanozymes

To elucidate the regulatory mechanism of NPs on mitochondrial respiratory chain complexes, we isolated hepatic mitochondria from different treatment groups and evaluated changes in the activities of Complexes I, III, IV, and V [7]. The experimental data demonstrated that compared with the sham‐operated group, the HIRI model group showed significant inhibition of all three complexes. The Fe_3_O_4_ intervention group did not substantially improve, whereas the CDs and FeCD treatment groups displayed partial recovery of enzymatic activity. Notably, the FeCD‐SS31 treatment group demonstrated the most pronounced increase in activity, with the activities of the complexes restored to near‐physiological levels. These findings provide molecular‐level evidence that FeCD‐SS31 effectively reverses mitochondrial electron transport chain dysfunction induced by HIRI (Figure 6A–D and Figure S19).

**FIGURE 6:**
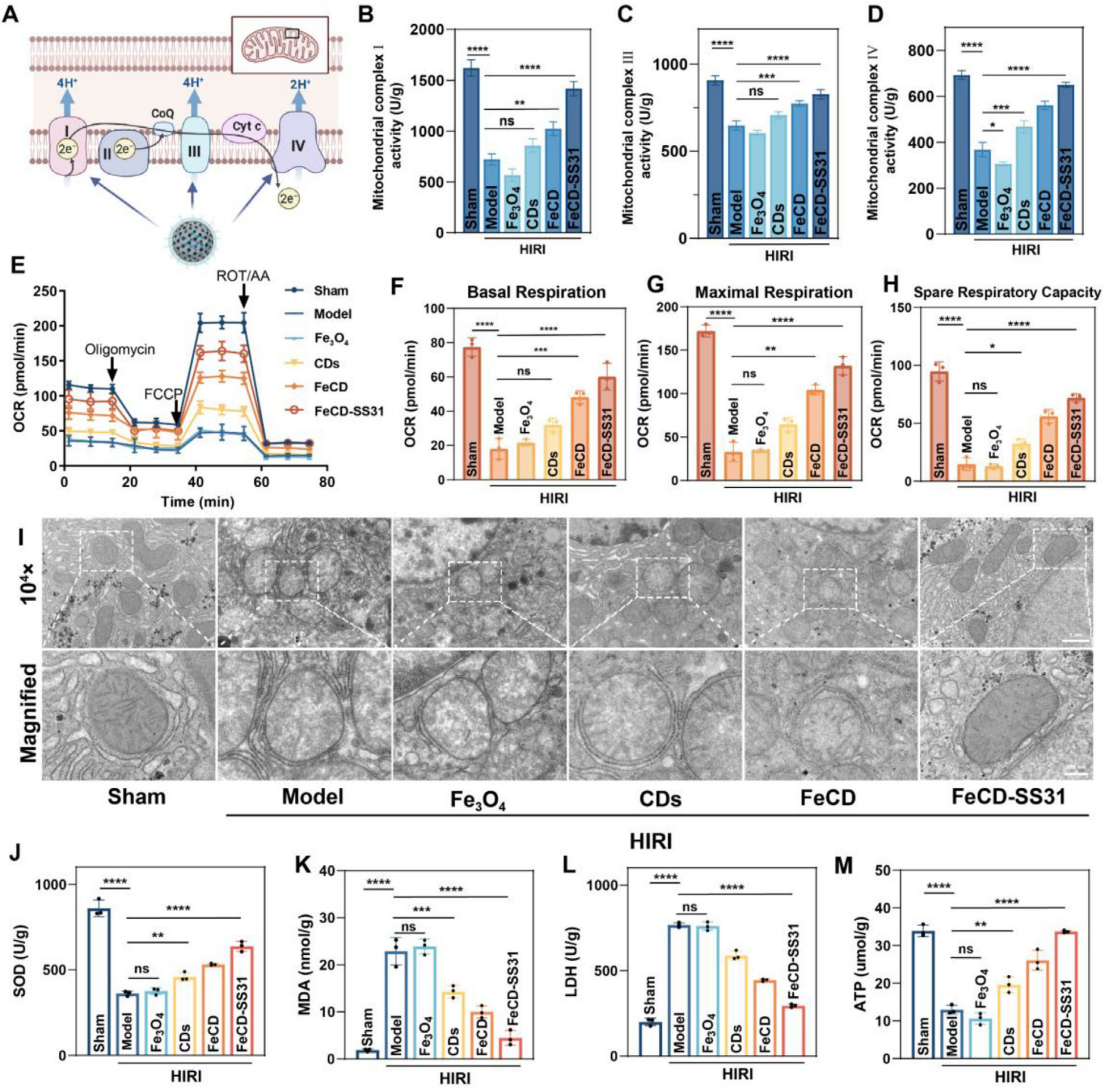
In vivo restoration of the mitochondrial respiratory chain by FeCD‐SS31 nanozymes. (A) Illustration of the mitochondrial respiratory chain. Activity of mitochondrial complexes I (B), III (C), and IV (D) following different treatments (*n* = 3). (E) OCRs of liver tissue after different treatments. Basal respiration (F), maximal respiration (G), and spare respiratory capacity (H) were determined based on the OCR results. (I) TEM images of hepatic mitochondria from different treatment groups (scale bar: 250 nm). SOD activities (J) and MDA (K), LDH (L), and ATP (M) levels in liver tissue homogenates from different treatment groups (*n* = 3). In panels (B–H), (J–M), the data are presented as the mean ± SD, and the *p*‐values were calculated by ANOVA. ∗ *p* < 0.05, ∗∗ *p* < 0.01, ∗∗∗ *p* < 0.001, ∗∗∗∗ *p* < 0.0001; ns, not significant.

Next, we employed a Seahorse XF metabolic analyzer to assess the oxygen consumption rate (OCR) of mouse liver tissue under the oxidative stress induced by HIRI [38]. Seahorse analysis revealed that the overall OCR of liver tissue cells in the HIRI group was significantly lower than that in the sham group, indicating impaired mitochondrial respiratory function due to HIRI. Treatment with Fe_3_O_4_ alone resulted in no notable improvement, whereas the CDs, FeCD, and FeCD‐SS31 treatment groups exhibited a progressive recovery trend (Figure 6E). Under basal conditions, basal respiration was significantly greater in the FeCD‐SS31 group than in the HIRI group (Figure 6F). Following FCCP stimulation, the maximal respiratory capacity of the HIRI group was markedly reduced compared with that of the control group, suggesting severe impairment of the mitochondrial electron transport chain reserve. In contrast, FeCD‐SS31 intervention substantially improved these parameters and significantly restored the spare mitochondrial respiratory capacity (Figure 6G,H). Furthermore, ATP‐linked respiration in the FeCD‐SS31‐treated group recovered to more than 85% of the level in the control group, demonstrating that the nanozymes effectively increased energy synthesis efficiency and reduced mitochondrial uncoupling (Figure S20).

TEM revealed distinct morphological differences among the groups. The HIRI group manifested characteristic mitochondrial pathology featuring crista reduction and structural disintegration, and the Fe_3_O_4_ group showed similar severe damage. In contrast, CDs and FeCD treatments progressively attenuated these defects, with FeCD providing greater protection. Notably, FeCD‐SS31 preserved ultrastructural integrity with well‐defined cristae, demonstrating effective protection against ischemia–reperfusion‐induced mitochondrial damage (Figure 6I).

HIRI disrupts the function of the mitochondrial electron transport chain, leading to increased electron leakage during electron transfer and a marked increase in •O_2_^−^ production, which subsequently depletes the activity of intracellular antioxidant enzymes. As shown in Figure 6J, SOD activity in liver tissue decreased significantly after HIRI, indicating that mitochondria‐derived oxidative stress overwhelmed the endogenous clearance capacity. Although Fe_3_O_4_ treatment failed to improve this condition, interventions with CDs, FeCD, and FeCD‐SS31 progressively restored SOD activity, suggesting that they mitigate •O_2_^−^ burden either by protecting respiratory chain function or through direct SOD‐mimetic activity. Electron leakage from the respiratory chain further induces mitochondrial lipid peroxidation, reflected by elevated malondialdehyde (MDA) levels (Figure 6K) [15], whereas mitochondrial DNA near the respiratory chain is more vulnerable to ROS attack, resulting in the accumulation of 8‐OHdG (Figure S21) [39]. The results of the assays revealed that the levels of MDA and 8‐OHdG tended to be consistent, with the levels of both markers significantly reduced in the FeCD‐SS31‐treated group.

Lactate dehydrogenase (LDH) serves as a key biomarker of oxidative stress‐induced cellular damage. Compared with that in the control group (200.63 U/g), LDH release was markedly elevated in hepatic tissues following HIRI induction (766.64 U/g). Treatment with CDs or FeCD notably attenuated LDH release, with FeCD‐SS31 having the most pronounced inhibitory effect (Figure 6L). ATP production is closely linked to mitochondrial functional and structural integrity. Accordingly, ATP levels were measured in liver tissues across different treatment conditions. The ATP content was significantly decreased in the HIRI group compared with that in normal tissue. In contrast, FeCD‐SS31 administration substantially alleviated ATP depletion in injured cells (Figure 6M).

To summarize, FeCD‐SS31 nanozymes effectively restore mitochondrial respiratory chain activity, increase bioenergetic efficiency, and reduce oxidative damage, collectively demonstrating their therapeutic potential in mitigating HIRI‐induced mitochondrial dysfunction and cellular injury.

### 2.7 Evaluation of the Therapeutic Efficacy of FeCD‐SS31 Nanozymes In Vivo

To investigate the therapeutic effect of our nanozyme on HIRI, a model in which C57BL/6J mice were used for experimental evaluation was established (Figure 7A). Hematoxylin and eosin (H&E) staining of liver tissue is presented in Figure 7B and provides direct morphological evidence of the treatment efficacy. The results revealed that the HIRI group exhibited significant hepatocellular swelling and degeneration, a disorganized lobular architecture, focal hepatocyte necrosis, and inflammatory cell infiltration, including that of neutrophils. The Fe_3_O_4_ treatment group showed no substantial improvement and still displayed extensive hepatocellular swelling, structural disarray, and even more pronounced inflammatory cell infiltration, which was possibly attributable to enhanced ·OH accumulation resulting from the peroxidase‐like activity of Fe_3_O_4_. In contrast, the CDs, FeCD, and FeCD‐SS31 treatment groups demonstrated progressively reduced liver damage, with the FeCD‐SS31 group showing nearly no inflammatory cell infiltration and tissue architecture closely resembling that of normal liver. ROS levels in liver tissues were detected by dihydroethidium (DHE) staining. The results showed that neither Fe_3_O_4_ nor CDs alone effectively scavenged ROS. In contrast, FeCD‐SS31 treatment markedly attenuated the DHE FL signal, indicating efficient ROS clearance in the liver (Figure 7B,D).

**FIGURE 7:**
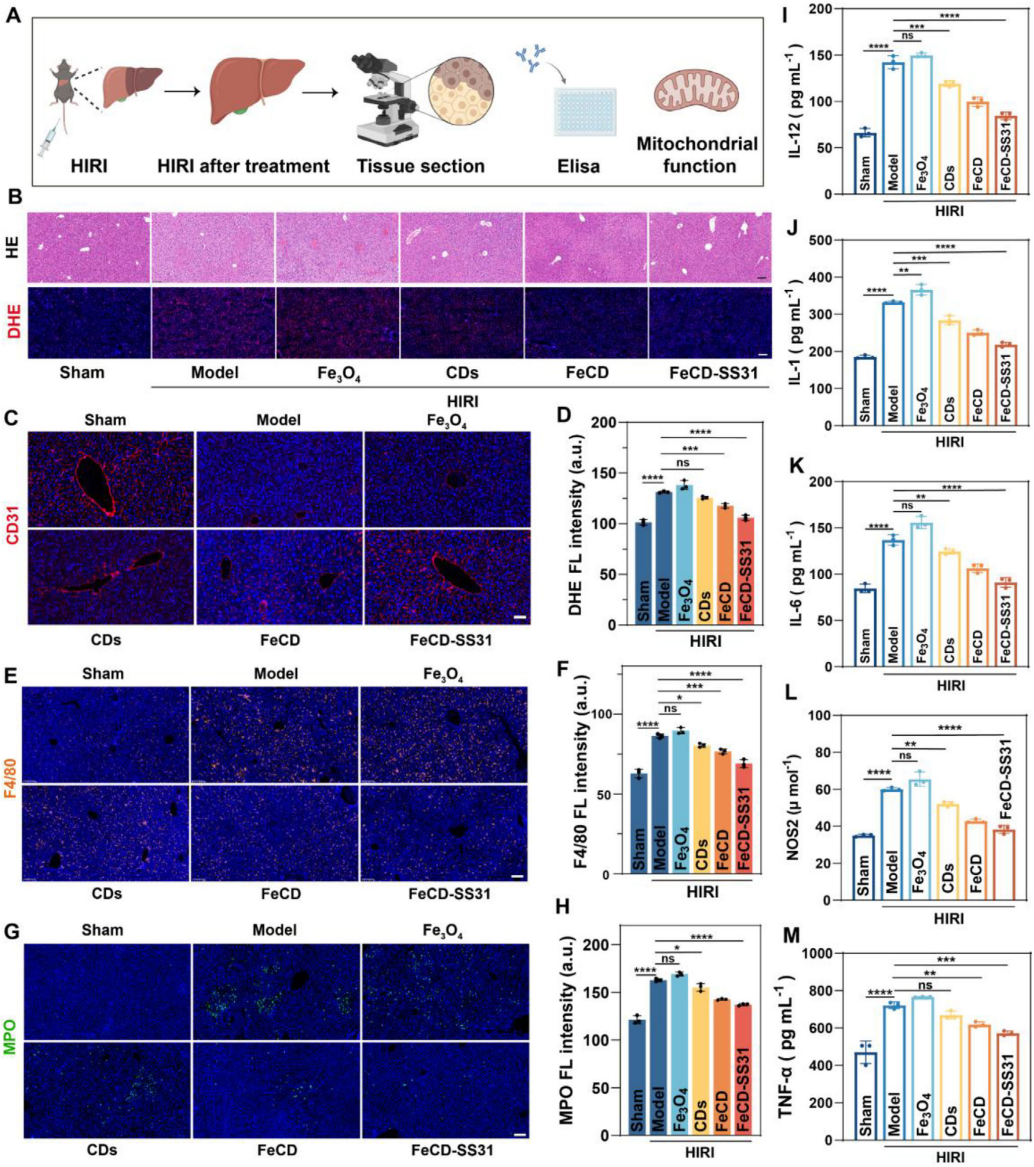
Evaluation of the therapeutic efficacy of FeCD‐SS31 nanozymes in vivo. (A) Schematic of HIRI model establishment. (B) H&E and DHE staining of liver tissues from different treatment groups (scale bar: 100 µm). (C) IF images of CD31 (scale bar: 50 µm). (D) Semiquantitative FL intensity analysis of DHE staining (*n* = 3). (E) F4/80 expression in liver tissue after different treatments (scale bar: 100 µm). (F) Semiquantitative FL intensity analysis of F4/80 staining (*n* = 3). (G) MPO expression in liver tissue after different treatments (scale bar: 100 µm). (H) Semiquantitative FL intensity analysis of MPO staining (*n* = 3). Measurement of serum IL‐12 (I), IL‐1 (J), IL‐6 (K), NOS2 (L), and TNF‐α (M) levels across treatment groups (*n* = 3). In panels (D,F,H–M), the data are presented as the mean ± SD, and the *p*‐values were calculated by ANOVA. ∗ *p* < 0.05, ∗∗ *p* < 0.01, ∗∗∗ *p* < 0.001, ∗∗∗∗ *p* < 0.0001; ns, not significant.

The pathophysiology of HIRI involves multiple mechanisms, among which the excessive generation of ROS is widely recognized as a pivotal damaging event that regulates diverse cellular phenotypes during the reperfusion phase. To investigate how FeCD‐SS31 modulates cellular phenotypes during ROS clearance, we performed IF staining of liver tissues using anti‐CD31 to label vascular endothelial cells (red), anti‐F4/80 to detect monocytes/macrophages (orange), and DAPI for nuclear staining (blue). As shown in Figure 7C,E,F, compared with the HIRI group, which exhibited marked activation of monocytes/macrophages and disrupted endothelial structure, the FeCD‐SS31 treatment group presented the lowest level of monocyte/macrophage activation and the best‐preserved vascular endothelial integrity. These results demonstrate that by efficiently scavenging excess ROS, FeCD‐SS31 effectively inhibited inflammatory cell infiltration and maintained the integrity of the hepatic microvascular endothelium within 6 h of treatment in the injury model.

Furthermore, we assessed liver function by measuring serum levels of ALT and AST (Figure S22). The results revealed that both the HIRI and Fe_3_O_4_ groups exhibited significantly higher ALT and AST levels than the normal range did. In contrast, the CDs, FeCD, and FeCD‐SS31 treatment groups showed a sequential reduction in these enzymes, with FeCD‐SS31 restoring levels closest to the normal range. This trend indicated a gradual recovery of liver function following treatment.

Activated monocytes/macrophages markedly increase the release of ROS and multiple proinflammatory cytokines [12]. To systematically evaluate the anti‐inflammatory effects of FeCD‐SS31, we measured the expression levels of key cytokines (IL‐1, IL‐6, IL‐12, NOS2 and TNF‐α) in serum from each group. As shown in Figure 7I–M, HIRI significantly elevated the expression levels of these cytokines, whereas FeCD‐SS31 treatment restored their levels to the normal range. Previous studies have indicated that IL‐1 stimulates neutrophils to release ROS and upregulates TNF‐α synthesis in Kupffer cells. As a key mediator of innate immunity, TNF‐α directly regulates the expression of IL‐6. Our results further demonstrate that FeCD‐SS31 effectively suppresses this signaling axis. Moreover, ischemia‒reperfusion injury induced the upregulation of the expression of NOS2, an enzyme that continuously generates nitric oxide and, under high oxidative stress, leads to excessive production of the highly cytotoxic peroxynitrite anion. Through its potent antioxidant activity, FeCD‐SS31 treatment blocked this cascade, thereby mitigating oxidative damage to the liver. In addition, we measured the serum levels of IL‐18 and IL‐1β (Figure S23), both of which serve as indirect indicators of pyroptosis. The results demonstrated that serum concentrations of HIRI significantly increased, whereas FeCD‐SS31 treatment reduced these levels to near‐normal ranges.

Neutrophils are key effector cells in acute injury and can be rapidly recruited to sites of inflammation within minutes [40]. To assess neutrophil infiltration in hepatic tissue, IF staining was performed on liver samples using an anti‐Ly6G antibody as a neutrophil‐specific marker. The results revealed pronounced neutrophil accumulation in HIRI mice, as clearly illustrated in Figures S24 and S25. In contrast, HIRI mice treated with FeCD‐SS31 showed minimal neutrophil infiltration at 6 h after surgery. Myeloperoxidase (MPO), a heme‐containing peroxidase predominantly expressed in neutrophils, was further measured to corroborate these findings. Livers from HIRI mice exhibited significantly elevated MPO levels, which is consistent with substantial neutrophil recruitment. In comparison, MPO activity in FeCD‐SS31‐treated HIRI mice remained comparable to that in healthy controls at 6 h after treatment, thereby supporting the reduction in neutrophil adhesion and infiltration observed via IF staining (Figure 7G,H).

In this study, using a C57BL/6J mouse model, we demonstrated that FeCD‐SS31 significantly alleviates HIRI. This nanozyme effectively scavenges intrahepatic ROS, improves liver function, suppresses inflammatory cell infiltration and activation, and decreases serum levels of multiple inflammation‐ and pyroptosis‐related cytokines. Collectively, these findings indicate that FeCD‐SS31 nanozymes mitigate ischemia–reperfusion‐induced hepatic tissue damage and inflammatory responses in vivo through synergistic multimechanistic actions.

### 2.8 In Vivo Imaging of HIRI and Preventive Efficacy in HIRI

#### 2.8.1 In Vivo Imaging of HIRI

Designed as a multifunctional nanozyme, FeCD‐SS31 was engineered to integrate both therapeutic and imaging capabilities. To validate its in vivo MRI performance, we established a murine HIRI model, administered either saline or FeCD‐SS31 intravenously, and dynamically monitored signal changes. The saline control group showed no significant imaging alterations throughout the observation period, and the IR regions were not delineated. In contrast, FeCD‐SS31 clearly outlined the injured areas within 10 min after injection. This phenomenon may be attributed to the impaired phagocytic function of Kupffer cells in the damaged region: normal hepatic tissue effectively takes up the NPs, generating a signal contrast that highlights IR zones during imaging. Over time, as the therapeutic effects of FeCD‐SS31 in the injured area increased, the corresponding signal intensity gradually diminished. This dynamic imaging pattern provides a novel radiological indicator for the noninvasive assessment of hepatic injury extent (Figure 8A,B). To confirm that the MRI signal changes were attributable to NPs accumulation rather than to edema or hemorrhage, we performed ICP‐OES to quantify iron content in liver tissues at 10 min post‐injection and correlated these data with T_2_ signal intensity in the left (injured) and right (non‐injured) lobes (Figure S26). The right lobe showed markedly higher iron content and a corresponding pronounced T_2_ signal decrease, while the left lobe exhibited only modest iron uptake and a slight signal increase. This contrast pattern reflects the negative contrast nature of Fe_3_O_4_: intact Kupffer cell function in the right lobe enabled substantial nanoparticle uptake and significant signal drop, whereas impaired phagocytosis in the injured left lobe limited nanoparticle accumulation, with possible edema/hemorrhage partially offsetting the modest signal decrease. These correlative data confirm that the observed MRI signal changes are dominated by NPs uptake in Kupffer cells, supporting FeCD‐SS31 as an effective MRI contrast agent for delineating hepatic injury.

**FIGURE 8:**
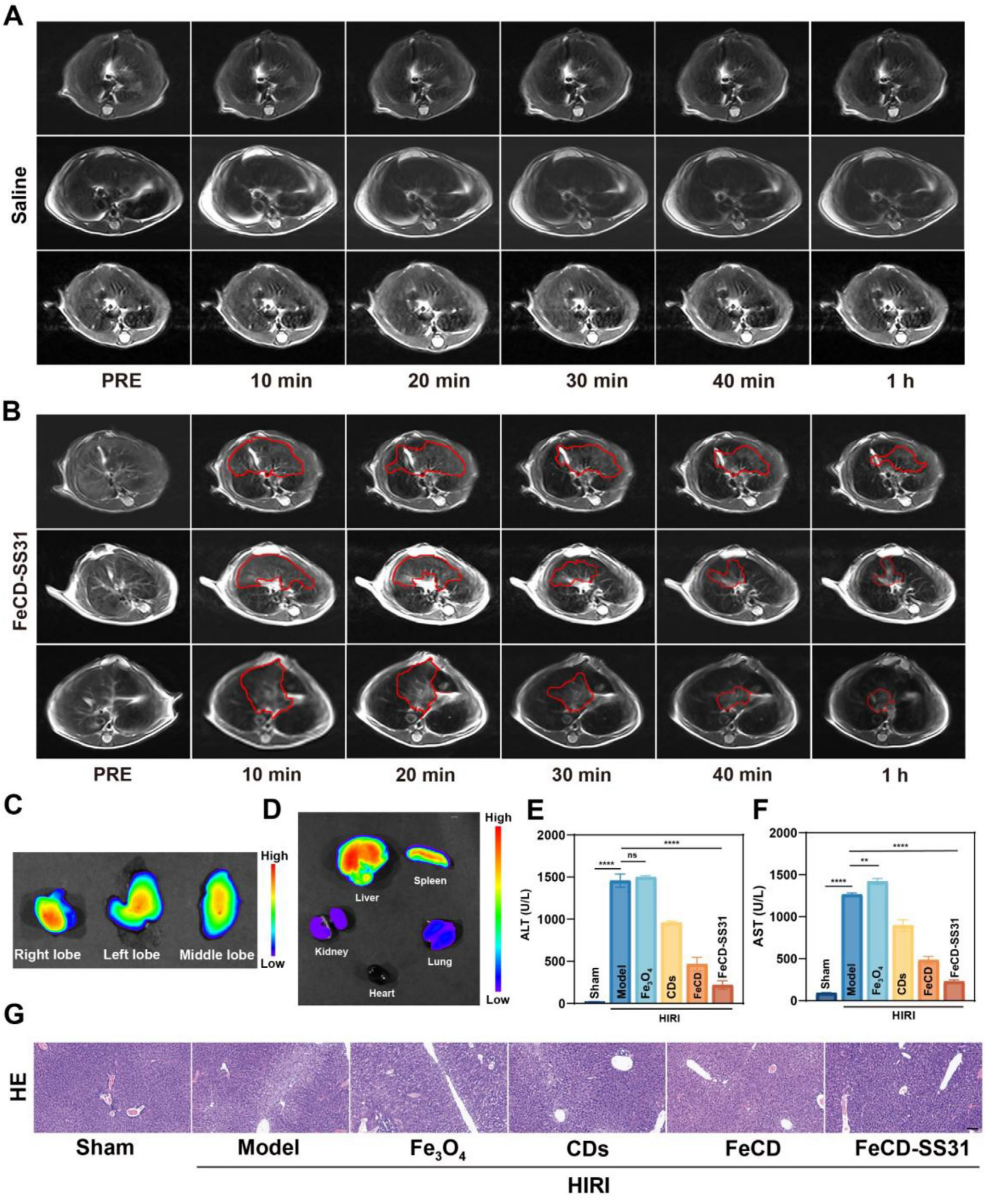
In vivo imaging of HIRI and preventive efficacy in HIRI. Representative MRI of HIRI mouse livers treated with saline (A) or FeCD‐SS31 (B) (*n* = 3). Representative FL images showing FeCD‐SS31@DiR accumulation in different lobes of HIRI livers (C) and major organs (D). Serum levels of ALT (E) and AST (F) following different treatments in the prevention model. (G) H&E staining of liver tissues from the HIRI prevention model following different treatments. In panels (E,F), the data are presented as the mean ± SD, and the *p*‐values were calculated by ANOVA. ∗ *p* < 0.05, ∗∗ *p* < 0.01, ∗∗∗ *p* < 0.001, ∗∗∗∗ *p* < 0.0001; ns, not significant.

Furthermore, we employed fluorescence imaging (FLI) to examine the distribution of NPs in the liver. The results demonstrated the most substantial NPs accumulation in the right hepatic lobe that had not undergone IR treatment, a distribution pattern consistent with the observations from MRI (Figure 8C and Figure S27A). Concurrently, the major organs collected after NPs injection were subjected to ex vivo FLI. The results indicated that the liver exhibited the greatest accumulation of NPs, demonstrating that the synthesized NPs can effectively target and accumulate in hepatic tissue, supporting their potential applicability for the treatment of liver diseases (Figure 8D and Figure S27B).

#### 2.8.2 Preventive Efficacy in HIRI

In addition to its therapeutic and diagnostic functions, this study further evaluated the preventive potential of FeCD‐SS31 by administering the nanoparticles intravenously 12 h prior to the surgical procedure, followed by the induction of HIRI. Liver tissues were then collected for histological examination via H&E staining (Figure 8G), and serum samples were obtained for liver function tests (Figure 8E,F). Both histopathological and biochemical analyses consistently demonstrated that FeCD‐SS31 effectively prevented ischemia‒reperfusion‐induced liver damage, suggesting its promising translational value as a preoperative prophylactic agent in hepatic surgery.

#### 2.8.3 In Vivo Pharmacokinetic Studies

To evaluate the pharmacokinetic profile of the drug, we conducted several pharmacokinetic studies. The circulation half‐life of FeCD‐SS31 was determined to be 1.77 h, indicating a relatively favorable blood retention profile. Regarding organ distribution, we monitored the real‐time FL signal in major organs at different time points post‐injection using an in vivo imaging system. Our results demonstrated that FeCD‐SS31 was predominantly distributed to the liver and spleen, with the liver signal peaking at approximately 1 h post‐injection, suggesting that the reticuloendothelial system serves as the primary clearance pathway for the NPs (Figures S28 and S29).

### 2.9 Biosafety of the FeCD‐SS31

The clinical translation of nanomaterials necessitates thorough biosafety evaluation. To assess the biocompatibility of FeCD‐SS31, we performed routine blood and blood biochemical index analyses in Kunming mice at multiple time points (0, 1, 7, 14, and 21 days) after administration, covering acute (1 day), short‐term (7 days), and long‐term (14 and 21 days) toxicity profiles (Figures S30 and S31). The results indicated no significant differences in blood parameters between the treated and control groups, suggesting the absence of systemic toxicity. Histopathological examination of major organs via H&E staining further confirmed the safety profile, revealing no notable morphological alterations in FeCD‐SS31‐treated animals compared with controls at any time point (Figure S32). Collectively, these results demonstrate favorable in vivo biocompatibility and support the translational potential of FeCD‐SS31 for clinical applications.

## 3 Limitations and Future Directions

While our findings demonstrate promising therapeutic efficacy, several limitations should be acknowledged, including the lack of systematic investigation into immune cell interactions, the incomplete characterization of global energy metabolic regulation, and the absence of direct comparisons with standard clinical regimens. Addressing these limitations will be a key direction for future research. Specifically, the interactions of FeCD‐SS31 with various immune populations and their signaling pathways warrant detailed exploration; the broader metabolic consequences, including glycolytic and fatty acid metabolism, remain to be fully elucidated. Beyond these aspects, future investigations should also focus on the long‐term biocompatibility and clearance profile of the nanozyme, as well as its potential immunogenicity upon repeated administration.

## 4 Conclusion

In summary, this study presents FeCD‐SS31, a theranostic nanozyme that treats HIRI through dual‐modal ROS modulation (SS31‐mediated mitochondrial repair and Fe_3_O_4_/CDs‐catalyzed ROS scavenging) and pyroptosis suppression (caspase‐1/IL‐1β/IL‐18 inhibition). In a HIRI mouse model, this integrated mechanism effectively improves liver function, reduces tissue damage, and mitigates systemic inflammation. Looking forward, the scientific and technological value of this system lies in its versatile design strategy: integrating diagnostic imaging, targeted mitochondrial repair, and dual‐modal ROS modulation, which can be readily adapted to other oxidative stress‐driven pathologies beyond HIRI, such as cardiovascular diseases, neurodegenerative disorders, and acute organ injuries. Thus, this platform not only offers a promising strategy for HIRI but also establishes a broadly applicable paradigm for combating oxidative stress‐related diseases.

## 5 Experimental Section

### 5.1 Synthesis of Various NPs

#### 5.1.1 Synthesis of Fe_3_O_4_ NPs

One gram of FeCl_3_·6H_2_O was dissolved in 30 mL of ethylene glycol, yielding a clear solution. Subsequently, 2.7 g of sodium acetate and 0.75 g of polyethylene glycol were added. The mixture was stirred vigorously for 30 min, then transferred into a 50 mL Teflon‐lined stainless‐steel autoclave and sealed. The autoclave was heated at 200°C for 8 h and then allowed to cool to room temperature. The resulting black product was washed repeatedly with ethanol and deionized water, followed by vacuum drying at 40°C for 24 h.

#### 5.1.2 Synthesis of Mesoporous Fe_3_O_4_ NPs

In a typical synthesis, 0.46 g of Fe_3_O_4_ NPs, 1 g of carbon precursor (chitosan), and 3 g of urea were dissolved in 40 mL of distilled water. The mixture was subjected to ultrasonic vibration for 20 min to form a homogeneous system and then transferred into a 50 mL Teflon‐lined autoclave. The autoclave was sealed and maintained at 200°C for 10 h, followed by cooling to room temperature. The product was separated using a magnet and repeatedly washed with deionized water and ethanol until the reddish‐brown supernatant became colorless and transparent. Finally, the product was dried in a vacuum oven at 40°C for 24 h to obtain mesoporous Fe_3_O_4_ NPs.

#### 5.1.3 Synthesis ***of** **PEG‐TK‐SS31***

A solution was prepared by dissolving 50 mg of PEG‐TK‐COOH, 5 mg of EDC, and 2 mg of NHS in 5 mL of DMSO. Activation of the carboxyl moieties on PEG‐TK‐COOH was achieved by stirring the reaction blend for 3 h at room temperature. Following this, 10 mg of SS31 was added, and the reaction was allowed to proceed with stirring for 12 h at room temperature. The product mixture was then dialyzed against deionized water (MWCO: 3500 Da) and freeze‐dried.

#### 5.1.4 Synthesis of CDs

A mixture of 1.2 g d‐biotin and 1.2 g glutathione was dissolved in 40 mL of formamide. The solution was then transferred into a Teflon‐lined autoclave and reacted at 160 °C for 8 h. After cooling to room temperature, the resulting product was dialyzed against deionized water using a dialysis membrane for 7 days. The solution was subsequently filtered through a 0.22 µm membrane, concentrated, and lyophilized.

#### 5.1.5 Synthesis of FeCD‐SS31

Iron oxide and CDs were vigorously stirred in an aqueous solution for 12 h. PEG‐TK‐SS31 was then added to the mixture, and stirring was continued for 24 h. The product was isolated using a magnet, repeatedly washed with deionized water and ethanol, and finally collected to obtain FeCD‐SS31.

#### 5.1.6 Synthesis of FeCD‐SS31@DiI and FeCD‐SS31@DiR

The synthesized FeCD‐SS31 was separately incubated with DiI and DiR dyes under constant stirring overnight, followed by centrifugation and washing to obtain FeCD‐SS31@DiI and FeCD‐SS31@DiR, respectively.

### 5.2 Cell Culture

Boster (Wuhan, China) supplied the AML‐12 cells (passages 8–12) used in this study. These cells were cultivated in DMEM/F12 containing 10% FBS, 1% penicillin‒streptomycin, 40 ng/mL dexamethasone, 10 µg/mL insulin, 5.5 µg/mL transferrin, and 5 ng/mL selenium. All experiments were conducted under controlled conditions (37°C, 5% CO_2_) to preserve cell viability.

### 5.3 Preparation of a HIRI Model in Mice

All animal procedures were approved by the Animal Ethics Committee of Chongqing Medical University (Approval No. (2025) 505) and conducted in accordance with institutional guidelines. Male C57BL/6 mice (6–8 weeks old) were obtained from the Experimental Animal Center of Chongqing Medical University and housed under standard conditions (22°C, 30%–70% humidity, 12 h light/dark cycle) with free access to food and water. To establish the HIRI model, the mice were fasted for 12 h prior to surgery. Under pentobarbital anesthesia, the abdominal area was shaved and disinfected with iodophor, and a midline laparotomy was performed. The portal vein and hepatic artery supplying the left and median liver lobes were isolated and occluded with a microvascular clamp for 60 min. During ischemia, the abdominal cavity was covered with PBS‐moistened gauze. Reperfusion was initiated by clamp removal, which was confirmed by the immediate restoration of blood flow (evident from color change) in the affected lobes.

### 5.4 Statistical Analysis

GraphPad Prism 9.5.1 (La Jolla, CA, USA) was used for statistical analysis. Data are presented as mean ± SD. The sample size (n) for each experiment is indicated in the corresponding figure legends. Differences among multiple groups were analyzed by one‐way ANOVA followed by Tukey's post‐hoc test. All applicable tests were two‐sided, and *p* < 0.05 was considered statistically significant (∗ *p* < 0.05, ∗∗ *p* < 0.01, ∗∗∗ *p* < 0.001, ∗∗∗∗ *p* < 0.0001; ns, not significant).

## Author Contributions

**Qianying Du**: conceptualization, methodology, Writing – original draft, Writing – review and editing, data curation. **Lian Xu**: conceptualization, methodology, writing – original draft, formal analysis. **Tingting Xia**: investigation, software, formal analysis, validation. **Wenli Zhang**: funding acquisition, visualization, resources. **Sijin Chen**: visualization, validation. **Xiyue Rong**: investigation, validation, data curation, resources. **Ying Luo**: validation, formal analysis, methodology, visualization. **Yi Wang**: software, formal analysis, methodology, data curation. **Jia Liu**: formal analysis, validation. **Yun Liu**: resources, project administration, visualization. **Haitao Ran**: methodology, validation, supervision. **Dajing Guo**: conceptualization, writing – original draft, writing – review and editing, funding acquisition, supervision. **Jie Xu**: conceptualization, methodology, funding acquisition, visualization, supervision, resources, project administration.

## Funding

This work was financially supported by the National Natural Science Foundation of China (grant nos. 82672504, 81901807, 82102063), the Natural Science Foundation of Chongqing (grant nos. CSTB2025NSCQ‐GPX0366, cstc2021jcyj‐msxmX0210), the Kuanren Talents Program of the Second Affiliated Hospital of Chongqing Medical University (grant nos. 2020–7 and 2021–24), and the Postgraduate Research and Innovation Projects of Chongqing Municipal Education Commission (grant no. CYS21222). Youth Project of Science and Technology Research Program of Chongqing Municipal Education Commission KJQN202300409. Postdoctoral Innovation Talents Support Program of Chongqing (CQBX202517).

## Ethics Approval and Consent to Participate

The animal experiments were approved by the Animal Ethics Committee of Chongqing Medical University and conducted in accordance with the guidelines of the Institutional Animal Care and Use Committee of Chongqing Medical University.

## Consent for Publications

All authors agreed to publish this research on journals.

## Conflicts of Interest

The authors declare no conflicts of interest.

## Supporting information




**Supporting File**: advs78277‐sup‐0001‐SuppMat.docx

## Data Availability

The data that support the findings of this study are available from the corresponding author upon reasonable request.
