# Supplementary material for: Mitochondria‐Targeting Iron–Carbon Nanozymes Combat Hepatic Ischemia–Reperfusion Injury via the Dual Mechanisms of Respiratory Chain Remodeling and Pyroptosis Inhibition With MRI Monitoring
Source: Adv Sci (Weinh). 2026 Oct 14:e78277. Online ahead of print. doi: 10.1002/advs.78277 (PMC13669213; doi:10.1002/advs.78277)
Supplement: Supplementary file 1 [file ADVS-9999-e78277-s001.docx]

**Support Information**

**Mitochondria-Targeting Iron–Carbon Nanozymes Combat Hepatic** **Ischemia–Reperfusion Injury via the Dual Mechanisms of Respiratory Chain Remodeling and Pyroptosis Inhibition with MRI Monitoring**

*Qianying Du^#^, Lian Xu^#^, Tingting Xia, Wenli Zhang, Sijin Chen, Xiyue Rong, Ying Luo, Yi Wang, Jia Liu, Yun Liu, Haitao Ran, Dajing Guo^*^ and Jie Xu^*^*

Q.D., L.X., T.X., W.Z., S.C., X.R., Y.L., Y.W., J.L., Y.L., D.G., J.X.

(1) Department of Radiology, Second Affiliated Hospital of Chongqing Medical University, Chongqing, 400010, P. R. China

(2) Key Laboratory of Intelligent Processing and Applications of Medical Imaging Big Data, Chongqing Municipal Health Commission, Chongqing, 400072, P. R. China

(3) Engineering Research Center for Fundamental and Translational Intelligent Molecular Imaging, Chongqing Municipal Education Commission, Chongqing, 400072, P. R. China

* Corresponding author:

E-mail: xj304847@cqmu.edu.cn (Jie Xu), guodaj@hospital.cqmu.edu.cn (Dajing Guo)

H.R.

Chongqing Key Laboratory of Ultrasound Molecular Imaging & Department of Ultrasound, Second Affiliated Hospital of Chongqing Medical University, Chongqing, 400010, P. R. China

#These authors contributed equally to this work.

**EXPERIMENTAL SECTION**

**Materials**

Iron (III) chloride (FeCl_3_) (I811935) was purchased from Macklin (Shanghai, China). Biotin (GC11194) and formamide (GC39381) were obtained from GLPBIO (Montclair, CA, USA). Phosphate-buffered saline (PBS) (PYG0021) was purchased from Boster (Wuhan, China). Dulbecco’s modified eagle medium/F12 (DMEM-F12) (ZYPYG0154), trypsin-EDTA solution (C0201), DAPI (C1005), Triton X-100 (P0096), and DiI (C1036) were purchased from Beyotime Biotechnology (Jiangsu, China). DiR (22070) was purchased from AAT Bioquest (CA, USA). ELISAs were purchased from MEIMIAN (Jiangsu, China). A Calcein-AM/PI Double Staining Kit (C542) was obtained from Dojindo Laboratories (Kumamoto, Japan). HMGB1 polyclonal antibody (10829-1-AP), CRT polyclonal antibody (10292-1-AP), CoraLited488-conjugated goat anti-rabbit IgG (H+L) (S00013-2) and CoraLited594-conjugated goat anti-rabbit IgG (H+L) (S00013-4) were obtained from Proteintech (Wuhan, China). An anti-GSDMD N-terminal antibody (DF13758) was obtained from Affinity (Jiangsu, China).

**Characterization of Different NPs**

The morphological characteristics of the synthesized NPs were examined using TEM (Talos F200X, USA) and SEM (HITACHI S4800, Japan). Elemental mapping for Fe, N, O, and C was conducted during TEM analysis. Zeta potentials of various NPs were measured with a Zetasizer Nano ZS90. XRD patterns were recorded on a BRUKER D8 ADVANCE diffractometer, and magnetic properties were assessed via VSM (LakeShore, USA). Brunauer–Emmett–Teller (BET) analysis was conducted with a Quantachrome Autosorb IQ3 apparatus (USA). The composition of the FeCD-SS31 was verified by FTIR (iS10 FT-IR spectrometer, USA) spectroscopy. To assess the EE and LC of CD in FeCD-SS31, a SpectraMax iD3 (USA) was utilized. XPS (Thermo Scientific K-Alpha, USA) of the FeCD-SS31 was also performed. ICP‒OES (Thermo ICP‒OES 7200, USA) was used to evaluate the EE values of Fe in FeCD-SS31. UV‒Vis spectrophotometry was used to determine the EE and LC of the FeCD-SS31 according to Equations 1 and 2.

**Equation 1.** EE (%) = (mass of CDs in NPs)/ (total initial mass of CDs) × 100%

**Equation 2.** LC (% w/w) = (mass of CDs in NPs)/ (total mass of FeCD-SS31) × 100%

We performed a dose-response release assay specifically to verify the responsive release of the SS31 peptide. Briefly, FeCD‑SS31 nanoparticles (with FITC‑labeled SS31) were encapsulated in dialysis bags and exposed to varying concentrations of H_2_O_2_ (0–200 µM) for the same duration. The fluorescence intensity of the external buffer was then measured to quantify the released FITC‑SS31.

**In Vitro MRI**

The in vitro MRI properties of FeCD-SS31 were assessed using a 3.0 T MRI scanner (MAGNETOM Prisma, Siemens, Germany). NP solutions spanning a range of concentrations (Fe: 0.00275–0.0275 mM) were imaged to determine the transverse relaxivity (r_2_). This was achieved by performing a linear regression of the inverse transverse relaxation time (1/T_2_) against the corresponding iron concentration.

**The cytotoxicity, intracellular endocytosis of FeCD-SS31 and the establishment of an in vitro HIRI model.**

*In Vitro Cytotoxicity Evaluation*

The cytotoxicity of the NPs was evaluated in AML-12 cells using a CCK-8 assay. Briefly, cells were seeded in 96-well plates and allowed to adhere overnight. The culture medium was subsequently replaced with fresh medium containing a series of concentrations (50–300 µg/mL) of Fe_3_O_4_, FeCD, or FeCD-SS31, followed by a 12-h incubation. Cell viability was then determined according to the manufacturer’s protocol for the CCK-8 assay.

*Intracellular Endocytosis*

AML-12 cells were seeded in confocal dishes or 6-well plates at a density of 1 × 10⁵ cells per dish/well and incubated overnight. For visualization, the cells in confocal dishes were incubated with DMEM/F12 medium containing FeCD-SS31@DiI (200 μg mL⁻¹) for various durations (0.5, 1, 2 or 4 h). Following incubation, fixation with 4% paraformaldehyde for 15 min and DAPI nuclear staining were performed. The resulting cellular uptake of FeCD‑SS31@DiI was imaged using CLSM (Nikon, Japan). For quantification, similarly treated cells grown in 6‑well plates were collected after incubation and subjected to FCM (FACS Vantage SE, Becton Dickinson, USA).

*Establishment of an In Vitro HIRI Model*

AML-12 cells were seeded in 12-well plates and allowed to adhere overnight. To establish the ischemia model, the culture medium was replaced with DMEM/F12 basal medium, and the cells were subjected to hypoxia for various durations (1, 2, 4, or 6 h). Cell viability was assessed after each interval using a live/dead staining assay to determine the optimal duration of hypoxia. Following this, a HIRI model was induced. After the predetermined ischemia period (4 h), the basal medium was replaced with complete culture medium to initiate reperfusion for different durations (1, 2, or 4 h). The extent of reperfusion injury was initially evaluated by live/dead staining. For quantitative analysis of cell death, apoptosis in the HIRI model was further assessed by FCM following Annexin V-FITC/PI staining.

**Analysis of In Vitro ROS Scavenging and Mitochondrial Targeting**

The elimination of •O^2^⁻ by FeCD-SS31 nanozymes was verified using ESR (Bruker EMXplus). In this assay, •O₂⁻ was generated with a xanthine/xanthine oxidase mixture. The nanozymes were then added and incubated with the radical solution for 5 or 10 min, after which the remaining •O^2^⁻ was captured by the spin trap DMPO. For SOD‑like kinetics, varying concentrations of xanthine were reacted with FeCD‑SS31 in a xanthine/xanthine oxidase‑nitroblue tetrazolium (NBT) system at 37 °C. The inhibition rates of NBT reduction were measured at 560 nm and fitted to the Michaelis‑Menten equation to obtain the apparent Kₘ.

The •OH-scavenging capacity of the FeCD-SS31 nanozymes was validated by ESR spectroscopy. The •OH radicals were generated using a TiO_2_/UV system (340 nm), which served as the control. The nanozymes were then introduced into the system and allowed to react for 5 or 10 min. Any residual •OH was subsequently detected by spin trapping with DMPO.

^1^O_2_-scavenging activity of FeCD-SS31 was also evaluated by ESR. ^1^O_2_ was generated by a photosensitizer under light, with or without the addition of FeCD-SS31 nanozymes. After incubation, the remaining ^1^O_2_ was trapped by TEMP, and the signal intensity of the resulting TEMP-^1^O_2_ adduct was measured by ESR.

The POD-mimetic activity of FeCD-SS31 was evaluated by measuring the catalytic oxidation of TMB in the presence of H_2_O_2_. The experimental groups included H_2_O_2_ + FeCD-SS31 + TMB, H_2_O_2_ + TMB, H_2_O_2_ + FeCD-SS31, and TMB alone, with final concentrations of 3% H_2_O_2_, 10 mg/mL FeCD-SS31, and 0.5% TMB, respectively, in a 200 μL system. The kinetic absorbance was recorded at one-minute intervals for 30 min after the addition of TMB using a UV‒Vis spectrophotometer. The steady-state kinetic assays for POD-like activity were performed using TMB as the substrate in the presence of H_2_O_2_. Typically, varying concentrations of TMB were incubated with FeCD‑SS31 nanozymes (final concentration: 0.5 mg/ml) and H_2_O_2_ (1M) in acetate buffer (pH 3.6) at 37 °C. The absorbance at 652 nm was monitored over time using a microplate reader. Initial reaction rates (V₀) were calculated from the linear portion of the absorbance versus time curves. The kinetic parameters (Kₘ and Vₘₐₓ) were obtained by fitting the data to the Michaelis‑Menten equation: V₀ = Vₘₐₓ × [S] / (Kₘ + [S]), using nonlinear regression. Turnover frequency (TOF) was calculated as TOF = Vₘₐₓ / [E], where [E] is the concentration of the Fe.

The mitochondrial targeting ability of FeCD-SS31 was investigated by assessing its colocalization with mitochondria. AML-12 cells seeded in confocal dishes overnight were treated with FITC-labeled FeCD-SS31 for 4 h. Afterward, the cells were incubated with MitoTracker Red and fixed with 4% paraformaldehyde, and the nuclei were stained with DAPI. Colocalization analysis of the green (FeCD-SS31-FITC) and red (mitochondria) signals was performed via confocal laser scanning microscopy.

To detect mitochondrial superoxide levels, AML-12 cells were seeded in confocal dishes and cultured overnight. Following the indicated treatments, the cells were stained with MitoSOX Red mitochondrial superoxide indicator, fixed with 4% paraformaldehyde, and counterstained with DAPI to label the nuclei. Images were subsequently acquired via CLSM.

To evaluate how effectively the NPs scavenge intracellular ROS, AML-12 cells were seeded in 12‑well plates for 24 h, then subjected to hypoxia and treated with various NPs. Total ROS levels inside the cells were measured using DCFH‑DA as a fluorescent probe. After a 15–25‑min incubation in darkness, the resulting fluorescence was quantified and observed under a fluorescence microscope.

**mRNA Sequencing Experimental Method**

*RNA Isolation and Library Preparation*

Following the manufacturer's protocol, TRIzol reagent (Invitrogen, CA, USA) was used to extract total RNA. RNA purity and concentration were measured with a NanoDrop 2000 spectrophotometer (Thermo Scientific, USA), and integrity was checked on an Agilent 2100 Bioanalyzer (Agilent Technologies, Santa Clara, CA, USA). Library construction was carried out using a VAHTS Universal V10 RNA-seq Library Prep Kit (Premixed Version) as per the manufacturer's instructions. OE Biotech Co., Ltd. (Shanghai, China) conducted the transcriptome sequencing and analysis.

*RNA Sequencing and Analysis of Differentially Expressed Genes*

Sequencing of the libraries on a DNBSEQ‑T7 platform produced 150 bp paired‑end reads, with roughly 23,876 raw reads per sample. After processing the raw fastq files with fastp, low‑quality reads were discarded, leaving about 22,878 clean reads per sample for subsequent steps. These clean reads were mapped to the reference genome using HISAT2. Read counts and FPKM values for each gene were derived via HTSeq‑count. To evaluate biological duplication among samples, PCA was carried out using R version 3.2.0.

Differential expression analysis was performed using DESeq2. A Q value <0.05 and a fold change >2 or <0.5 were set as the thresholds for significantly differentially expressed genes (DEGs). Hierarchical cluster analysis of DEGs was performed using R (v 3.2.0) to determine the expression patterns of genes in different groups and samples. A radar map of the top 30 genes was constructed to show the expression of up- or downregulated DEGs using the R packet ggradar.

Using R (v 3.2.0), enrichment analyses of the DEGs were performed based on the hypergeometric distribution across GO, KEGG pathway, Reactome, and WikiPathways to identify significantly enriched terms. The same software was then used to generate column, chord, and bubble diagrams for those terms.

**Mechanisms of FeCD-SS31 in the Treatment of HIRI In Vitro and In Vivo**

*In Vitro and In Vivo Investigation of Pyroptosis Inhibition by FeCD‑SS31*

We performed IF staining for HMGB1, CRT, and cleaved caspase-1 to verify the inhibition of the canonical pyroptosis pathway by the nanozymes. First, AML-12 cells were cultured in separate confocal dishes and incubated for 12 h. Subsequently, all groups except the sham group were subjected to hypoxia. Afterward, they were treated with Fe_3_O_4_, CDs, FeCD, or FeCD-SS31. Following three washes with PBS, the cells were fixed in 1.5 mL of 4% paraformaldehyde for 15 min, blocked with 5% BSA for 45 min, and then incubated overnight at 4 °C with primary antibodies against CRT, HMGB1, or cleaved caspase-1. After another PBS wash, the cells were exposed to the corresponding fluorescent secondary antibodies for 1 h. Finally, nuclei were counterstained with DAPI, and imaging was performed using CLSM.

Culture supernatants were collected from cells subjected to the same treatment as those used for CLSM and were tested for concentrations of IL-18, IL-1β, TNF-α, IFN-γ, IL-1, IL-12, and IL-6 using corresponding commercial ELISA kits in accordance with the manufacturers' instructions.

At the animal level, liver tissues were first collected from the sham-operated, HIRI, and FeCD-SS31-treated groups for WB analysis of caspase-1and GSDMD expression. IF assays for CRT, HMGB1, and cleaved caspase-1 were subsequently performed on liver tissues from the respective treatment groups.

*In Vivo Restoration of the Mitochondrial Respiratory Chain by FeCD-SS31 Nanozymes*

To evaluate mitochondrial respiratory function, we first isolated mitochondria from liver tissue samples of equal weights and measured the activities of Complexes I, III, and IV using commercial assay kits. To determine the OCR, we employed a Seahorse XF metabolic analyzer. First, liver tissues from mice subjected to different treatments were collected, and the cellular OCR was subsequently measured using an XF-24 flux analyzer. During the assay, the liver tissue samples were first treated with 2.0 μM oligomycin to assess ATP production across groups. Next, 1 μM FCCP was added to measure the rate of increase in mitochondrial oxygen consumption, which was defined as the maximal mitochondrial respiratory capacity. Finally, 0.5 μM antimycin A and 0.5 μM rotenone were introduced to inhibit the mitochondrial respiratory chain, thereby completely suppressing mitochondrial oxygen consumption.

Furthermore, mitochondrial ultrastructure was examined by TEM. To quantify bioenergetic output, ATP levels were measured in homogenized liver tissues using a commercial kit. Finally, the oxidative stress status was evaluated by determining the activity of SOD and the concentrations of MDA and LDH in tissue homogenates.

**Evaluation of the Therapeutic Efficacy of FeCD-SS31 Nanozymes In Vivo**

A mouse model of HIRI was established as previously described. The animals were randomly allocated into six groups (including a sham-operated group) and treated with different NPs (Fe_3_O_4_, CDs, FeCD, or FeCD‑SS31). Six hours after treatment, liver tissues were collected for the following analyses: H&E staining of paraffin sections and IF staining for DHE, CD31, MPO, Ly6G and F4/80. Serum samples were also collected to measure ALT and AST levels, and the concentrations of IL-1, IL-18, IL-1β, IL-6, TNF-α, and IL-12 were determined using ELISA kits.

**In Vivo Imaging of HIRI and Preventive Efficacy in HIRI**

*In Vivo* *MRI*

To validate the in vivo MRI capability of FeCD-SS31, a murine HIRI model was established using above described method. The model mice were administered either saline or FeCD-SS31 via tail vein injection. Dynamic T_2_-weighted MR scans of the liver were subsequently performed at 10, 20, 30, and 40 min, as well as at 1 h after injection. The T_2_WI parameters were set as follows: TR = 7500 ms; TE = 85 ms; slice thickness = 0.9 m; FOV = 80 mm; and ﬂip angle = 150°.

*In Vivo FLI*

FLI was conducted with a near-infrared fluorescence (NIRF) imaging system (NightOWL II LB983, Germany) at excitation/emission wavelengths of 748/780 nm. In the HIRI model, FeCD-SS31@DiR was administered intravenously (40 mg/kg), after which the mice were euthanized, and the livers were harvested (1 h posttreatment). The distribution and accumulation of NPs across different hepatic lobes were subsequently examined. To further evaluate the systemic biodistribution of the NPs, FeCD-SS31@DiR was injected into healthy mice, which were allowed to circulate for 24 h. Following this period, the animals were euthanized, and major organs were collected for ex vivo fluorescence imaging.

*Preventive Efficacy in HIRI*

The mice were randomly assigned to six experimental groups: (1) Sham, (2) Model (HIRI), (3) Fe_3_O_4_ + HIRI, (4) CDs + HIRI, (5) FeCD + HIRI, and (6) FeCD-SS31 + HIRI. All the NPs were administered intravenously at a dosage of 40 mg/kg 6 h prior to the surgical procedure. Following 12 h of hepatic HIRI induction, blood samples were collected for biochemical assessment. Liver function was evaluated by measuring serum levels of ALT and AST. Additionally, the left liver lobes were harvested for histological analysis via H&E staining.

*In Vivo Pharmacokinetic Studies*

For pharmacokinetic evaluation, FeCD‑SS31 was intravenously administered to mice at a dose of 40 mg/kg. To determine the circulation half‑life, blood samples were collected at predetermined time points post‑injection, and the plasma iron content was measured to determine the drug concentration. The half‑life was derived by fitting the concentration‑time data to a one‑compartment pharmacokinetic model. For organ biodistribution analysis, mice were sacrificed at different time intervals, and major organs (heart, liver, spleen, lungs, and kidneys) were harvested. The fluorescence signal in each organ was captured using an in vivo imaging system to assess the distribution pattern over time.

**Biosafety of the FeCD-SS31**

The in vivo biosafety of FeCD-SS31 was evaluated in 6–8-week-old male Kunming mice. The animals were randomly divided into five groups: a control group and four experimental groups, which were euthanized at 1, 7, 14, and 21 days after injection of the NPs. Each mouse received FeCD-SS31 intravenously at a therapeutic dose (40 mg/kg). At each designated time point, blood was collected for routine hematological and biochemical analysis, and major organs were harvested for histological examination by H&E staining.

**Figure S1**.VSM of the FeCD-SS31.

**Figure S2**.XPS survey spectra of FeCD-SS31

**Figure S3**. XPS spectra of N1s with peak fitting.

**Figure S4.** Dose-response curve for ROS-triggered SS31 release (n=3).

**Figure S5**. In vitro T2 -weighted MRI of FeCD-SS31 at 3.0 T MRI scanner.

**Figure S6**. The r2 values of the FeCD-SS31 at 3.0 T MRI scanner.

**Figure S7.** POD-like activity of FeCD-SS31 nanozymes: FeCD-SS31 nanozymes can oxidize TMB from colorless to blue oxTMB in the presence of H_2_O_2_.

**Figure S8.** Michaelis-Menten kinetics analysis of FeCD-SS31 with TMB as the substrate (n=3).

**Figure S9.** The apparent Michaelis constant (Kₘ) of FeCD‑SS31, with xanthine as the substrate (n=3).

**Figure S10.** GSEA of pyroptosis‑related pathways.

**Figure S11.** GO enrichment analysis of mitochondrial‑related functional categories.

**Figure S12.** The quantitative analysis of HMGB1 expression in AML-12 cells upon different treatments. Data are represented as mean ± SD (n = 3). All the *p* values were calculated by ANOVA. * *p* < 0.05, ** *p* < 0.01, *** *p* < 0.001, **** *p* < 0.0001, ns, not significant.

**Figure S13.** The quantitative analysis of CRT expression in AML-12 cells upon different treatments. Data are represented as mean ± SD (n = 3). All the *p* values were calculated by ANOVA. * *p* < 0.05, ** *p* < 0.01, *** *p* < 0.001, **** *p* < 0.0001, ns, not significant.

**Figure S14.** The quantitative analysis of cleaved caspase-1 expression in AML-12 cells upon different treatments. Data are represented as mean ± SD (n = 3). All the *p* values were calculated by ANOVA. * *p* < 0.05, ** *p* < 0.01, *** *p* < 0.001, **** *p* < 0.0001, ns, not significant.

**Figure S15.** The concentrations of IFN-γ (A), IL-12 (B) and IL-1 (C) quantified in the cell culture supernatants following different treatments (n = 3). Data are represented as mean ± SD. All the *p* values were calculated by ANOVA. * *p* < 0.05, ** *p* < 0.01, *** *p* < 0.001, **** *p* < 0.0001, ns, not significant.

**Figure S16.** The quantitative analysis of caspase-1 expression in liver tissue upon different treatments. Data are represented as mean ± SD (n = 3). All the *p* values were calculated by ANOVA. * *p* < 0.05, ** *p* < 0.01, *** *p* < 0.001, **** *p* < 0.0001, ns, not significant.

**Figure S17.** (A) WB analysis of GSDMD and GSDMD-N in vivo. The quantitative analysis of GSDMD (B) and GSDMD-N (C) expression in liver tissue upon different treatments. Data are represented as mean ± SD (n = 3). All the *p* values were calculated by ANOVA. * *p* < 0.05, ** *p* < 0.01, *** *p* < 0.001, **** *p* < 0.0001, ns, not significant.

**Figure S18.** IF images of CRT expression in liver tissue after different treatments (scale bar: 100 μm).

**Figure S19.** Activity of mitochondrial complexes Ⅴ following different treatments. Data are represented as mean ± SD (n = 3). All the *p* values were calculated by ANOVA. * *p* < 0.05, ** *p* < 0.01, *** *p* < 0.001, **** *p* < 0.0001, ns, not significant.

**Figure S20.** ATP production determined based on the OCR results (n=3). Data are represented as mean ± SD. All the *p* values were calculated by ANOVA. * *p* < 0.05, ** *p* < 0.01, *** *p* < 0.001, **** *p* < 0.0001, ns, not significant.

**Figure S21.** 8-OHdG levels released from liver tissues across different experimental groups (n=3). Data are represented as mean ± SD. All the *p* values were calculated by ANOVA. * *p* < 0.05, ** *p* < 0.01, *** *p* < 0.001, **** *p* < 0.0001, ns, not significant.

**Figure S22.** Measurement of serum ALT (A) and AST (B) levels in different treatment groups (n=3). Data are represented as mean ± SD. All the *p* values were calculated by ANOVA. * *p* < 0.05, ** *p* < 0.01, *** *p* < 0.001, **** *p* < 0.0001, ns, not significant.

**Figure S23.** The concentrations of IL-1β (A) and IL-1β (B) quantified in the mice serum following different treatments (n = 3). Data are represented as mean ± SD. All the *p* values were calculated by ANOVA. * *p* < 0.05, ** *p* < 0.01, *** *p* < 0.001, **** *p* < 0.0001, ns, not significant.

**Figure S24.** IF images of Ly6G expression in liver tissue after different treatments (scale bar: 100 μm).

**Figure S25.** Corresponding quantitative analysis of Ly6G expression (n = 3) Data are represented as mean ± SD. All the *p* values were calculated by ANOVA. * *p* < 0.05, ** *p* < 0.01, *** *p* < 0.001, **** *p* < 0.0001, ns, not significant.

**Figure S26.** (A) T2‑weighted MR signal intensity in liver (n = 3). (B) Iron content in liver tissues (n = 3). Data are represented as mean ± SD. All the *p* values were calculated by ANOVA. * *p* < 0.05, ** *p* < 0.01, *** *p* < 0.001, **** *p* < 0.0001, ns, not significant.

**Figure S27.** Corresponding quantitative analysis of FL images of the liver lobes (A) and major organs (B) removed from mice (n=3). Data are represented as mean ± SD. All the *p* values were calculated by ANOVA. * *p* < 0.05, ** *p* < 0.01, *** *p* < 0.001, **** *p* < 0.0001, ns, not significant.

**Figure S28.** ICP-MS analysis of Fe in the blood at different time intervals post the FeCD-SS31 injection. Data are represented as mean ± SD (n = 3).

**Figure S29.** Representative FL images showing the biodistribution of FeCD‑SS31@DiR in major organs at different time intervals.

**Figure S30.** Routine blood examination results of Kunming mice after intravenous injection of the FeCD-SS31 NPs at different time points.

**Figure S31.** Blood biochemical examination of Kunming mice after intravenous injection of the FeCD-SS31 NPs at different time points.

**Figure S32.** H&E staining images of the major organs (scale bar: 100 μm).
